# Strong broadband intensity noise squeezing from infrared to terahertz frequencies in lasers with nonlinear dissipation

**DOI:** 10.1515/nanoph-2025-0259

**Published:** 2025-09-19

**Authors:** Sahil Pontula, Jamison Sloan, Nicholas Rivera, Marin Soljačić

**Affiliations:** Department of Physics, MIT, Cambridge, MA 02139, USA; Department of Electrical Engineering and Computer Science, MIT, Cambridge, MA 02139, USA; Research Laboratory of Electronics, MIT, Cambridge, MA 02139, USA; E. L. Ginzton Laboratory, Stanford University, Stanford, CA 94305, USA; School of Applied and Engineering Physics, Cornell University, Ithaca, NY 14853, USA; Department of Physics, Harvard University, Cambridge, MA 02138, USA

**Keywords:** squeezed light, nonlinear loss, intensity noise squeezing

## Abstract

The generation and application of squeezed light have long been central goals of quantum optics. Intensity noise squeezing of bright (coherent) states (“bright squeezing”), in contrast to squeezed vacuum, is relatively underdeveloped. The current state of the art has generally been restricted to narrow operating wavelength ranges and does not natively support strong intracavity and broadband output squeezing. Here, we show how lasers with sharp intensity-dependent dissipation can support strong intensity noise squeezing from infrared (IR) to terahertz (THz) wavelengths, the latter of which has eluded quantum light generation. Our protocol realizes strongly ( >10 dB) intensity noise-squeezed intracavity quantum states as well as output squeezing surpassing gigahertz bandwidths. Furthermore, we show how the same systems also support self-pulsing and bistability, enabling control of light in both the mean field and noise domains. Our protocol could enable advances in low-noise communication, cavity QED, and quantum sensing across the electromagnetic spectrum.

## 1 Introduction

The generation of states of light with noise “squeezed” below the standard quantum limit for a coherent state is a decades-old pursuit of quantum optics. In these squeezed states, the variance in one observable (such as amplitude or phase) is reduced at the expense of another, permitting levels of quantum fluctuations, which lie below the standard quantum limit. Such squeezed states of light have been harnessed for continuous variable quantum computing as well as precision sensing and metrology [1], [2]. The most common methods to generate squeezed light employ laser-pumped nonlinear crystals. For example, subthreshold optical parametric amplifiers have been used to produce up to 3 dB of intracavity squeezing [3], [4], [5] and 15 dB of propagating squeezed vacuum [6], [7], [8].

By contrast, schemes to generate squeezing in bright states of light (specifically, intensity noise squeezing of macroscopic coherent states) are less mature, despite their promise as probes for precision measurement and spectroscopy [1], [9], [10], [11], [12], [13], [14], [15], [16]. We will refer to this type of squeezing as “bright squeezing” going forward, not to be confused with bright squeezed vacuum [17]. Established methods to generate bright squeezing include displacement of squeezed vacuum, lasing using a squeezed vacuum reservoir and parametric or periodic driving, second harmonic generation, Kerr nonlinearity in fiber-optic interferometers, and “quietly pumped” semiconductor lasers [18], [19], [20], [21], [22], [23], [24], [25]. However, these methods leave the field of bright squeezing underdeveloped in the following ways. First, strong intracavity bright squeezing is not native to the aforementioned methods, owing to the 3 dB intracavity squeezing limit in parametric amplification schemes and relaxation oscillation noise in lasers. If realized, intracavity squeezed coherent states would have exciting potential applications in qubit nondemolition readout in cavity QED, polaritonic chemistry, optomechanical cooling, quantum metrology, and enhanced light–matter interactions [5], [26], [27], [28], [29], [30]. Second, large bandwidths are not readily accessible with existing bright squeezing methods (e.g., due to GHz relaxation oscillation noise), limiting their application in high-speed applications such as quantum radar and optical computing/communication. Finally, existing methods to produce intense squeezed states have been generally limited to narrow wavelength ranges in the visible and near-infrared (e.g., due to nonlinear phase matching and conversion efficiency constraints). As a result, there are large wavelength ranges (MIR-THz) in which intensity squeezing has never been demonstrated, despite tantalizing applications in quantum-enhanced chemical fingerprinting, wireless communication, and solid-state qubit manipulation [31].

These wavelengths spanning from the IR to the THz have been particularly well-served by semiconductor lasers, owing to their wide gain bandwidths, scalable form factors, and ease of electrical pumping. Several methods have been explored to produce intensity squeezing directly from semiconductor lasers, including so-called “quiet pumping” (pump noise suppression) and optical feedback/dispersive loss to exploit amplitude-phase correlations [32], [33], [34]. However, these methods do not produce intracavity squeezing and generally only achieve output squeezing at low noise frequencies, leaving the large excess noise from so-called “relaxation oscillations” at higher frequencies unmitigated. Thus, the majority of modern lasers do not surpass – or even reach – the shot noise limit at large bandwidths. This, together with the limitations of other nonlinear optical techniques described above, highlights a broad open challenge in producing sources of highly squeezed intense light that are versatile in wavelength and bandwidth.

Here, we show how lasers equipped with Kerr nonlinearity and frequency-dependent outcoupling can enable sharply nonlinear intensity-dependent dissipation and act as a source of intense squeezed light from IR to THz wavelengths, reducing intracavity intensity fluctuations to more than 10 dB below the shot noise limit and strongly squeezing output fluctuations over GHz bandwidths. Our approach exploits intensity-dependent dissipation, in conjunction with a semiconductor gain medium, to create a laser architecture that natively produces light with intensity fluctuations far below the shot noise limit. We show that semiconductor laser architectures are aptly suited for this purpose due to their compact form factor, strong intrinsic optical nonlinearities, and ease of on-chip integration with the low loss resonators and photonic crystals required to generate frequency-dependent dissipation. In addition, we explain how these same architectures can exhibit classical nonlinear phenomena such as self-pulsing and bistability. Together, these functionalities could pave the way toward combined temporal and quantum noise control over light across the electromagnetic spectrum.

## 2 Theory of nonlinear dispersive loss

**Figure 1:**
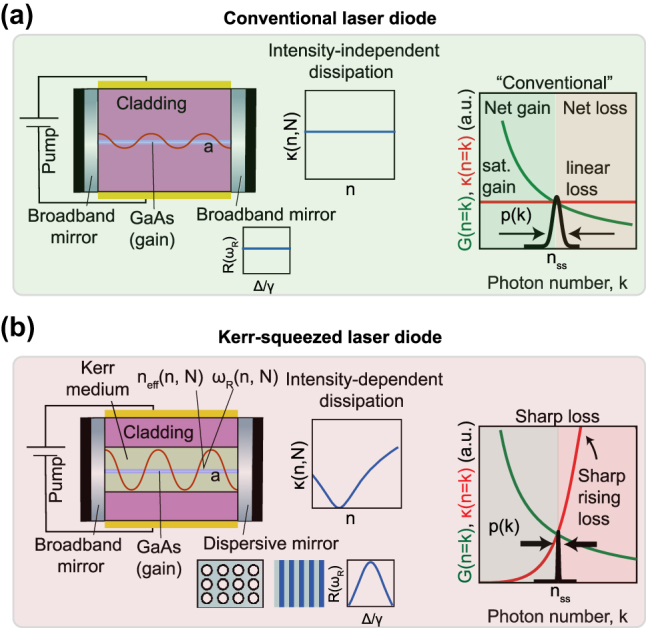
Comparison of semiconductor lasers with and without nonlinear dissipation. (a) Basic semiconductor laser diode heterostructure design with normal (linear) dissipation. The mirrors are broadband and lack the sharp frequency-dependent reflection profile needed to create nonlinear loss. (b) Analogous laser with nonlinear dispersive loss. Dispersive outcoupling is generated via the sharp frequency-dependent transmission of a photonic crystal element. Coupling of Kerr nonlinearity from the Kerr material with a dispersive mirror of reflectivity *R*(*ω*_*R*_) creates sharp nonlinear loss *κ*(*n*, *N*) (where *n*, *N* denote the photon and carrier numbers, respectively). Here, Δ = *ω*_*R*_ − *ω*_*d*_ denotes detuning from the dispersive resonance centered at *ω*_*d*_ and *γ* denotes the bandwidth of the resonance. Under sharp loss, the steady state photon probability distribution *p*(*k*) (mean *n*_ss_, variance Δ*n* ^2^) is squeezed $\left(\Delta n^{2}/n_{\text{ss}}\ll 1\right)$ compared to that of a conventional laser $\left(\Delta n^{2}/n_{\text{ss}}\geq 1\right)$ . The steady state photon number is determined by the location of intersection between saturable steady state gain *G*(*n*=*k*) and loss *κ*(*n*=*k*). The variance of the probability distribution is determined by the effective “steepness” of intersection of the gain and loss curves.

We first describe how, under the right conditions, the combination of Kerr nonlinearity and frequency-dependent loss lead to a laser cavity with an effective *intensity-dependent loss* that controls the quantum state of light produced by the laser. A typical laser, such as that shown in Figure 1a, has an intensity-independent loss that leads to coherent state statistics when pumped well above threshold. Consider now the cavity architecture shown for a semiconductor laser in Figure 1b. For clarity of notation, we will use $\hat{X}$ to denote an arbitrary quantum mechanical operator, $X\equiv \left\langle \hat{X}\right\rangle$ to denote the mean field value of this operator, and *X*_ss_ to denote its steady state mean field value. We focus on a single cavity mode, with annihilation operator $\hat{a}$ . As is well known, a cavity containing a Kerr nonlinearity develops an intensity-dependent resonance frequency due to the intensity-dependent index of the Kerr material [35]. In the case of semiconductor lasers, free carrier nonlinearities also shift the cavity resonance. Then, the cavity resonance frequency depends linearly on the photon number and inverted carrier density *n* and *N* as

(1) $$\omega_{R}\left(n,N\right)=\omega_{0}\cdot \left(1+\beta n+\sigma N\right),$$

as derived in the SI. This form for the cavity resonance shift due to semiconductor nonlinearities has been analyzed previously using coupled mode theory and supported experimentally [36], [37], [38], [39]. Here, *ω*_0_ is the bare resonance frequency of the cavity mode $\hat{a}$ , *β* is a dimensionless per-photon nonlinearity that can be directly calculated from the Kerr nonlinear coefficient *n*_2_ or nonlinear susceptibility *χ* ^(3)^, and the carrier nonlinearity *σ* is material-dependent and is directly related to the linewidth enhancement factor *α*_*L*_ (see SI for details).

Additionally, in the laser cavity of Figure 1b, one of the end facets is a sharply dispersive element, such as a Fano resonance structure or a Bragg reflector, which equips the cavity with sharply frequency-dependent dissipation through its reflection coefficient *R*(*ω*_*R*_). When combined, the intensity-dependent resonance frequency and frequency-dependent dissipation give the cavity mode an effective *intensity-dependent dissipation*, which can promote the formation of quantum states [40], [41]. The one critical assumption for this description is that the temporal response of the dispersive mirror is fast compared to the round trip time of the cavity. This corresponds to an adiabatic limit where the dispersive resonance, which sets the cavity transmission *T*(*ω*_*R*_), is able to near-instantaneously follow shifts in the cavity frequency caused by the nonlinearities. When these assumptions are fulfilled, the cavity field is subject to an effective intensity-dependent damping rate

(2) $$\begin{array}{cc} \kappa \left(n,N\right)\equiv \kappa \left(\omega_{R}\left(n,N\right)\right) & =-FSR\cdot \log R\left(\omega_{R}\left(n,N\right)\right)\\ & \approx FSR\cdot T\left(\omega_{R}\left(n,N\right)\right), \end{array}$$

where FSR denotes the free spectral range (inverse of cavity round trip time) and the approximation in the second line holds when *R*(*ω*_*R*_) ≈ 1. Sharply frequency-dependent reflectivity profiles enable the dissipation rate *κ*(*n*, *N*) to take on forms that are highly nonperturbative in *n*, making this type of nonlinear dissipation fundamentally different than the types of nonlinear dissipation realized by multiphoton absorption. One example of such a reflectivity profile has been realized in self-pulsing Fano lasers [42] with low mode volumes, which, when augmented with a Kerr nonlinear material, could create strongly nonlinear dissipation. As we will show, systems exhibiting this kind of loss can provide new behaviors not just in their steady states but also through new quantum noise behaviors.

Note that in Figure 1b, we consider a semiconductor laser with separate gain and Kerr nonlinear elements. We choose to use a different material for the Kerr nonlinearity in order to avoid possible dispersive resonant effects of optical nonlinearity near transition energies in the gain material. The Kerr material is chosen to be a GaAs-based semiconductor due to its strong optical nonlinearity from bound carriers. Semiconductor lasers with nonlinear dispersive loss based on “active nonlinearity” (in which the gain and Kerr materials are the same) may be possible, but the timescale of resonant effects may call into question the adiabatic assumption of the cavity resonance frequency’s instantaneous response to changes in photon number, thus placing such systems outside the scope of the models we consider here.

## 3 Laser dynamics under nonlinear dispersive loss

Semiconductors typically fall into the category of so-called “class B” lasers, in which the polarization dynamics decay quickly relative to the timescales associated with carrier recombination and cavity decay. In this case, the polarization dynamics are adiabatically eliminated, resulting in Heisenberg–Langevin equations for photon number and carrier number operators, as derived in the SI. In the mean field, these equations read

(3a) $$\dot{n}=\left(G\left(n,N\right)-\kappa \left(n,N\right)\right)n,$$

(3b) $$\dot{N}=I-\left(nG\left(n,N\right)+\gamma_{\|}N\right).$$

To be maximally general here, we allow the gain *G* and loss *κ* to depend on both the carrier density *N* and photon number *n* (e.g., saturable gain is photon number-dependent). In writing this form of the gain and loss, we have assumed that the gain and loss respond effectively instantaneously to changes in the photon and carrier number. Pumping is performed by carrier injection using current *I* (in units of carrier density per unit time), and *γ*_‖_ denotes the nonradiative decay rate of carriers. The case of optically pumped excitation of free carriers is described in the SI.

In all examples presented in the main text, we consider linear gain, which neglects saturation effects, so that *G*(*n*, *N*) = *G*(*N*) = *G*_*N*_(*N* − *N*_trans_) with *N*_trans_ the transparency carrier density and *G*_*N*_ the equivalent gain cross-section [43]. We found no phenomenological differences using logarithmic quantum well gain or including the effects of gain saturation [44]. Effects of two photon absorption and varying linewidth enhancement factor are explored in the SI.

## 4 Noise properties under nonlinear dispersive loss

The noise properties of semiconductor lasers can be computed by considering operator valued fluctuations of the Heisenberg–Langevin equations from their mean field solutions. In the steady state, this results in a pair of coupled linear equations for the operator values fluctuations $\delta \hat{n}$ and $\delta \hat{N}$ (see SI for derivation), which are given as:

(4) $$\begin{array}{cc} \left[\begin{array}{c} \delta \dot{\hat{n}}\\ \delta \dot{\hat{N}} \end{array}\right] & =\left[\begin{array}{cc} -n_{\text{ss}}\kappa_{n} & n_{\text{ss}}\left(G_{N}-\kappa_{N}\right)\\ -\kappa_{\text{ss}} & -\left(n_{\text{ss}}G_{N}+\gamma_{\|}\right) \end{array}\right]\left[\begin{array}{c} \delta \hat{n}\\ \delta \hat{N} \end{array}\right]\\ & \;+\left[\begin{array}{c} {\hat{F}}_{n}-n_{\text{ss}}\kappa_{\omega }{\hat{F}}_{\phi }\\ {\hat{F}}_{N} \end{array}\right]. \end{array}$$

Here, *κ*_ss_ denotes the steady state loss (which equals steady state gain), *κ*_*n*_ ≡ ∂*κ*/∂*n* = (∂*κ*/∂*ω*)(∂*ω*/∂*n*) = *βω*_0_ *κ*_*ω*_ represents the sharpness of the dispersive loss with respect to photon number, and *κ*_*N*_ ≡ ∂*κ*/∂*N* = (∂*κ*/∂*ω*)(∂*ω*/∂*N*) = −*α*_*L*_ *G*_*N*_ *κ*_*ω*_/2 = *σω*_0_ *κ*_*ω*_. Further, ${\hat{F}}_{n},{\hat{F}}_{N},{\hat{F}}_{\phi }$ are zero-mean Langevin forces associated with the photon number, carrier, and phase number equations of motion (respectively), whose correlators give all sources of noise in the system. All partial derivatives are evaluated at steady state.

Because of the macroscopic intensities we consider in this work, operator-valued fluctuations are well approximated by Gaussian statistics that can be calculated through the linearized Heisenberg–Langevin equation formalism, where $\sqrt{\left\langle \delta {\hat{X}}^{2}\right\rangle }\ll \left\langle \hat{X}\right\rangle$ for an arbitrary observable $\hat{X}$ . More exotic non-Gaussian quantum states such as mesoscopic Fock states may be accessible using nonlinear dissipation in highly nonlinear systems (see SI).

Important physical parameters to characterize intensity noise are the relaxation oscillation frequency and damping rate of relaxation oscillations. As derived in the SI, these can be calculated from the complex poles of the fluctuation dynamics:

(5) $$\begin{array}{cc} \Omega_{R}^{2} & \approx \left(n_{\text{ss}}G_{N}+\gamma_{\|}\right)\left(n_{\text{ss}}\kappa_{n}\right)+n_{\text{ss}}\left(G_{N}-\kappa_{N}\right)\kappa_{\text{ss}},\\ \Gamma_{1} & \approx n_{\text{ss}}\left(G_{N}+\kappa_{n}\right)+\gamma_{\|}. \end{array}$$

These measures provide an important way to understand the effect of nonlinear dispersive loss on quantum noise. They will also dictate mean field dynamics that result from fluctuations from steady state operation.

## 5 Mean field dynamics

**Figure 2:**
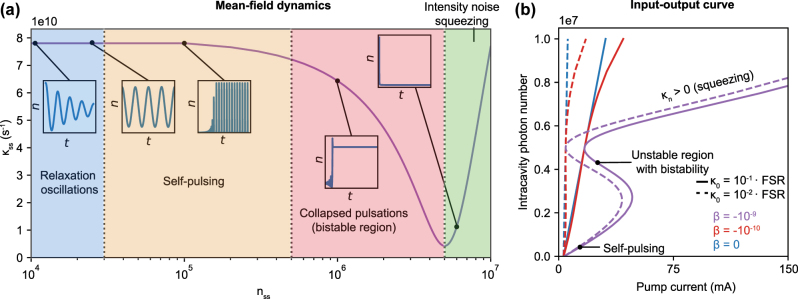
Mean field dynamics and steady state behavior. (a) Dynamical and steady state solutions in semiconductor lasers with nonlinear dispersive loss. In the region *κ*_*n*_ = ∂*κ*/∂*n* < 0 (photon numbers left of the Fano resonance), a variety of different behaviors are possible. At large detunings (small initial photon number *n*_init_, blue region), the loss does not depend strongly on photon number, and the relaxation oscillations typical of conventional semiconductor lasers are observed. At a certain detuning (*n*_init_), the relaxation oscillations become critically damped and, at smaller detunings, they become undamped, leading to self-sustained picosecond pulses (orange region). When the pump enters the bistable region (red region), the pulses become transient and the laser ultimately collapses to a continuous wave (CW) steady state. Lastly, to the right of the loss minimum (green), relaxation oscillations are heavily damped since *κ*_*n*_ = ∂*κ*/∂*n* > 0, leaving a strongly squeezed CW steady state. Plots were produced by considering a transient increase in intracavity intensity by 5 % at *t* = 0 relative to *n*_init_ = *n*(*t* = 0). (b) Steady state intracavity photon number *n*_ss_ as a function of pump current (S-curve) for two different linear background losses *κ*_0_ and three nonlinear strengths *β*. The indicated unstable region is bypassed by the bistable point and is not generally accessible during lasing. In these simulations, we use parameters based on experimentally determined values for buried heterostructure lasers with GaAs gain and AlGaAs cladding (Figure 1b): cavity length *L* = 0.5 mm, cavity volume *V* = 10^−16^ m^3^, bare cavity resonance frequency *ω*_0_ = 2.16 × 10^15^ s^−1^ (873 nm, GaAs bandgap), transparency density *N*_trans_ = 10^24^ m^−3^, nonradiative decay rate *γ*_‖_ = 10^8^ s^−1^, linear gain coefficient *G*_*N*_ = 1/*V* ⋅ d*G*/d*N* = 7,388 s^−1^, and linewidth enhancement factor *α*_*L*_ = 2.5 [43]. The Fano resonance is centered at photon number *n*_*c*_ = 5 × 10^6^, and its width is *γ* = 10^13^ s^−1^.

We begin by considering the mean field steady state and dynamical solutions that emerge for a Kerr nonlinear semiconductor laser with a symmetric Fano resonance (Figure 1b, nonlinearity and mode profile simulated in SI), with transmission *T*(*ω*_*R*_) = Δ^2^/[Δ^2^ + *γ* ^2^/4], where Δ = *ω*_*R*_ − *ω*_*d*_ and *γ* is the FWHM of the resonance. The center frequency of the resonance is designed to lie at *ω*_*d*_ = *ω*_*R*_(*n*_*c*_) so that the cavity experiences minimum loss at photon number *n*_*c*_. As shown in Figure 2a, the mean field behavior can differ drastically depending on the loss *κ*_ss_ = *κ*(*n* = *n*_ss_) and its sharpness $\kappa_{n}=\left(\partial \kappa /\partial n\right){|}_{n=n_{\text{ss}}}$ .

The mean field dynamics of the equations of motion allow diverse modes of operation, as shown in Figure 2a. The key driving force for these behaviors is the variation in the damping rate for relaxation oscillations (Eq. (5)), which describes relaxation back to the mean field steady state. We plot the temporal evolution of the intracavity photon number following a transient 5 % increase in the photon number at *t* = 0 relative to the initial steady state. For *κ*_*n*_ ≈ 0 (low *n*_ss_ and far detuned from Fano resonance, blue region), relaxation oscillations are observed. For *κ*_*n*_ ≪ 0, the relaxation oscillations become critically damped and eventually undamped (orange region), resulting in oscillations that transition into self-generated and self-sustained pulses. The pulses are quenched when the initial photon number enters the bistable region’s lowest branch, ultimately collapsing to the topmost branch (and bypassing the intermediate unstable branch). For *κ*_*n*_ > 0, relaxation oscillations are strongly damped (Γ_1_ grows with *κ*_*n*_ in Eq. (5)). We note that in many semiconductor lasers that are not operated very far above threshold, intensity noise is often far from the shot noise limit due to the relaxation oscillation peak. The nonlinear loss in the region *κ*_*n*_ > 0 suppresses this peak by over 40 dB, as we will show. Physically, the nonlinear loss magnifies the strength of attraction of the laser steady state (a fixed point of the rate equations) in proportion to the slope *κ*_*n*_. This has the effect of strongly resisting deviations from the steady state photon number, leading to the strong intensity squeezing described in the next section.

Self-pulsing has been reported previously using photonic crystal-based “Fano” lasers with saturable free carrier absorption from a nanocavity [42]. Here, we see that a similar phenomenon occurs due to a different physical mechanism: the combination of Kerr nonlinearity and dispersive loss. Suppose that the laser is pumped to a CW steady state lying in the self-pulsing region of Figure 2a. A transient increase in intracavity intensity (e.g., due to spontaneous emission into the lasing mode) now decreases the photonic loss, providing a positive feedback mechanism that builds up the intracavity intensity further. This should continue up to the point where the stimulated emission rate is high enough to drop the carrier density below threshold. The pump then builds up the carrier density again, and the pulsing continues. Further details about the self-pulsing behavior, including an analysis of the pulse profile, are provided in the SI.

We now examine the steady state input–output curve (S-curve), as shown in Figure 2b. Linear loss presents an intensity-independent loss profile and leads to the well-known linear dependence of steady state photon number on pump current (as well as clamping of the carrier density and gain above threshold). In the presence of dispersive loss, moderate nonlinearity (*β* = −10^−10^) begins to modify the steady state behavior. For pump currents just above threshold, the behavior is close to linear. However, as the pump current increases, so does the loss, pulling down the input–output curve to a sublinear behavior. For even stronger nonlinearity (*β* = −10^−9^), a bistable transition occurs that creates a range of photon numbers, which have no stable steady state solution (“unstable region”). In particular, this occurs because there is a nonzero photon number at which the cavity experiences minimum loss. The topmost bistable branch (with *κ*_*n*_ > 0) needs to be accessed hysteretically “from above,” by pumping to a high power (beyond the right bistable edge) and slowly lowering the power. As we will see, just above the left point of bistability on the topmost bistable branch, *n*_ss_ stays approximately constant while the photon number variance Δ*n* ^2^ can decrease sharply, resulting in strong intensity noise squeezing.

While our focus is on the application of nonlinear dissipation to semiconductor lasers in this paper, we note that nonlinear dissipation potentially admits a much larger class of laser and amplifier architectures that can realize the mean field dynamics described here, not limited to semiconductor gain media. The essential elements are (1) Kerr nonlinearity and (2) dispersive loss, which together can endow any cavity in principle with nonlinear dissipation. This mechanism also marks a departure from works using saturable absorption to generate self-pulsing [45] because it may not require large pump powers – the loss can be engineered to be minimized at a nonsaturating photon number. This enables self-pulsing, for example, in our system to emerge at modest pump strengths. More generally, through different dispersive loss profiles, arbitrary shapes of intensity-dependent dissipation can be generated, enabling one to engineer behaviors different from those of saturable absorbers. For instance, the sharp loss profiles we consider here mimic “super-saturable absorption” and could enable narrower pulses at comparable intensities to normal saturable absorption. We also note that a future direction enabled by our work is the intersection of self-pulsing and intensity noise squeezing (below) to generate “squeezed pulsing” from IR to THz frequencies.

## 6 Broadband intensity noise squeezing

We now describe how the mechanism of intensity-dependent loss can compress steady state photon statistics. The steady states of all lasers are characterized by a balance between saturable gain and loss. In a conventional laser with “linear loss,” the loss rate seen by the cavity field is the same for all photon numbers, that is, the loss is independent of *n*. For photon numbers where gain exceeds loss, an effective “force” encourages occupation of yet higher photon numbers; for photon numbers where loss dominates gain, an effective force encourages occupation of lower photon numbers. The intersection point where “gain equals loss” represents the equilibrium point between these two forces, and consequently determines the mean photon number of the cavity in the laser steady state (Figure 1). While the intersection point determines the mean photon number, the behavior of the photon number-dependent gain and loss in the vicinity of this intersection dictates the variance of the steady state photon number probability distribution *p*(*k*). In conventional lasers, which are far above threshold, the probability distribution approaches that of a coherent state, with Poissonian statistics (Figure 1a).

This situation changes significantly when linear loss is replaced by a strongly intensity-dependent loss. If the loss rises sharply with photon number around its intersection with the saturable gain, then the steady state probability distribution becomes compressed compared to the case of linear loss. Intuitively, this is because the disparity between loss and gain around the steady state is magnified relative to the conventional laser, resulting in larger “forces” that squeeze the probability distribution to sub-Poissonian statistics (Figure 1b). Roughly speaking, the photon number variance is determined by the ratio of the slopes of the gain and loss. This mechanism enables the sharp loss laser to create steady states with variance lower than the mean, a feature only possible in nonclassical light. In the most extreme limit, the loss may rise so sharply that only a single number state (the mean) has a substantial probability of occupation, approaching a cavity Fock state. However, realizing intracavity Fock states would likely require systems with fewer photons and stronger nonlinearities, such as exciton–polariton condensates [40].

To quantify this effect in semiconductor laser systems, we consider the photon number variance, given by ${\left(\Delta n\right)}^{2}=\frac{1}{\pi }\int_{0}^{\infty }d\omega \delta n^{2}\left(\omega \right)$ , where $\delta n^{2}\left(\omega \right)=\left\langle \delta {\hat{n}}^{†}(\omega )\delta \hat{n}\left(\omega \right)\right\rangle$ gives the spectrum of (intracavity) intensity fluctuations (see SI) and is governed by the Fourier transform of Eq. (4). A useful parameter to quantify the quantum nature of light is the Fano factor, defined as *F* = (Δ*n*)^2^/*n*_ss_. The Fano factor is 1 for Poissonian light, corresponding to the shot noise limit; values below one indicate sub-Poissonian light below the shot noise level. We calculate the most general expression for *F* (including carrier nonlinearity) in the presence of nonlinear dispersive loss in the SI. For weak Kerr and carrier nonlinearities, *F* → 1 when pumping far above threshold, approaching Poissonian (coherent) statistics. Our main result here is that for strong Kerr nonlinearity, the Fano factor behaves as

(6) $$F\to \kappa_{\text{ss}}/\left(n_{\text{ss}}\kappa_{n}\right)$$

for large *n*_ss_. This equation naturally provides a guide for the requirements of an experimental demonstration to realize strong squeezing (*F* ≪ 1) by our mechanism, namely low background loss (high intrinsic *Q* factor), high photon number (as in a laser cavity), and sharp dependence of the nonlinear outcoupling on photon number (e.g., via strong Kerr nonlinearity and dispersive outcoupling via a photonic crystal as we consider here).

**Figure 3:**
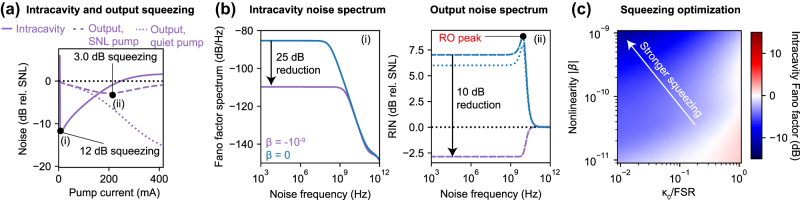
Intensity noise squeezing. (a) Squeezing in intracavity intensity noise (including pump noise) as well as output intensity noise (with and without pump noise, evaluated at 1 MHz noise frequency) over a range of pump currents. “SNL” pump denotes a shot noise limited pump, whereas “quiet pump” indicates no pump noise (e.g., due to constant current driving). Here, *β* = −10^−9^ and *κ*_0_ = 10^−2^ ⋅ FSR. (b) Intracavity Fano factor spectrum (*δn* ^2^(*ω*)/*n*_ss_) and output relative intensity noise (RIN) as a function of noise frequency for the two points indicated in (a). Blue curves represent a “conventional” laser with identical, but linear, loss (solid for SNL pump, dotted for quiet pump). Relaxation oscillations are absent in (i) due to the low loss. The purple dashed line in (ii) indicates a laser with nonlinear dissipation and with an SNL pump, following the convention of (a). (c) Intracavity squeezing increases with stronger nonlinearity and lower background loss. In these figures, system parameters are the same as those in Figure 2.

In Figure 3, we demonstrate the effects of intensity noise squeezing in semiconductor lasers with nonlinear dissipation. Figure 3a plots intracavity and output squeezing for the upper bistable branch where *κ*_*n*_ > 0 (the noise in the lower branch resembles that of conventional semiconductor lasers with enhanced relaxation oscillation noise). For linear loss (shown in SI), the Fano factor *F* → 1 (shot noise limit) far above threshold. The behavior of Fano factor for nonlinear dispersive loss is phenomenologically different. The noise behavior resembles that for linear loss when the detuning from the Fano resonance grows large (*κ*_*n*_ ≈ 0). Approaching the left bistable edge, the cavity frequency approaches the Fano resonance and, for a certain *n*_ss_, Eq. (6) approaches a minimum, corresponding to maximum intracavity squeezing. The Fano factor does not decrease indefinitely due to intensity-carrier noise coupling and finite carrier noise from nonradiative decay processes. Nonetheless, low linear background losses, sharp dispersive dissipation, and large Kerr nonlinearities can create intracavity squeezing over 10 dB below the shot noise limit at pump strengths near threshold (owing to the system’s bistability). This last feature alleviates the strong pump requirements of many squeezing schemes and could be significant for many quantum sensing and computing applications requiring low intensity.

Output intensity noise can be computed from intracavity noise by coupled mode theory, with special care taken for the dispersive nature of the loss (see SI). In Figure 3a, we see that output squeezing occurs for larger pump strengths compared to those required for intracavity squeezing, since the outcoupling loss must be large enough to enable destructive interference between the outcoupled field and external vacuum fluctuations (dashed purple curve). We also note that nonlinear dissipation can be combined with quiet pumping schemes to yield even stronger output squeezing. However, output squeezing through quiet pumping alone becomes significant further above threshold (dotted purple curve).

We now proceed to examine the intracavity and output intensity noise spectra. Figure 3b demonstrates the drastic reduction in intracavity intensity noise *δn* ^2^(*ω*)/*n*_ss_ as well as the large-bandwidth ( >10 GHz) squeezing in output intensity noise accessible through nonlinear dissipation. The large squeezing bandwidth is enabled through relaxation oscillation (RO) suppression, which is not possible with quiet pumping schemes alone (blue dotted curve). Due to the sharp loss, the RO peak is in general significantly suppressed compared to the case of linear loss (mathematically, the RO frequency and damping rate are both increased in accordance with Eq. (5)).

Finally, in Figure 3c, we show how intracavity squeezing can be optimized through minimizing linear background losses (e.g., material absorption) and maximizing the dimensionless Kerr coefficient (through, for example, low mode volumes or materials with stronger *χ* ^(3)^ response).

We conclude this section by noting that different photonic crystal structures may permit sharper dispersive losses and thus stronger squeezing. One example is a distributed Bragg reflector (DBR), whose loss profile and noise properties we consider in the SI (Figures S3 and S4).

## 7 IR and terahertz squeezing using quantum cascade lasers

To emphasize the generality of the physics of nonlinear dissipation, we now apply our mechanism to quantum cascade lasers (QCLs), showing that strong intensity squeezing can be extended to spectral ranges where quantum light is difficult to obtain, such as the mid-IR and THz, but is nonetheless tantalizing for applications including low-noise broadband communication as well as spectroscopic chemical fingerprinting [31]. Theoretical proposals to use pump noise suppression in interband cascade lasers have been made [46], together with experimental applications of injection locking and optical feedback to reduce intensity noise below that of free-running QCLs [47], [48], [49]. Here, we demonstrate how our physical mechanism enables (1) strong intracavity intensity noise squeezing of a bright coherent state and (2) broadband output noise squeezing below the shot noise limit in the mid-IR and THz, both of which have not been accessible by other methods. QCLs employ intersubband transitions for stimulated emission, allowing recycling of the carrier population and, therefore, high output powers, since a single carrier can now generate *m* photons if *m* gain stages are used [50], [51]. This endows QCLs with giant intrinsic Kerr nonlinearities [52], [53] that have been employed in a variety of applications, such as frequency comb generation for molecular spectroscopy in the infrared [54]. We note that the picosecond timescale of these nonlinearities can fulfill the adiabaticity criterion for nonlinear dispersive loss [55] and that low-loss dispersive mirrors have been previously used to create dispersion-compensated QCL frequency combs [56]. Strongly intensity noise-squeezed light from QCLs, if realized, is extremely promising given that (1) intensity noise squeezing is more difficult to achieve in QCLs than other semiconductor lasers due to nonradiative decay of carriers in multiple levels [57], and (2) QCLs operate at wavelengths that are of great interest for sensing and communications but are inaccessible by most other lasers.

**Figure 4:**
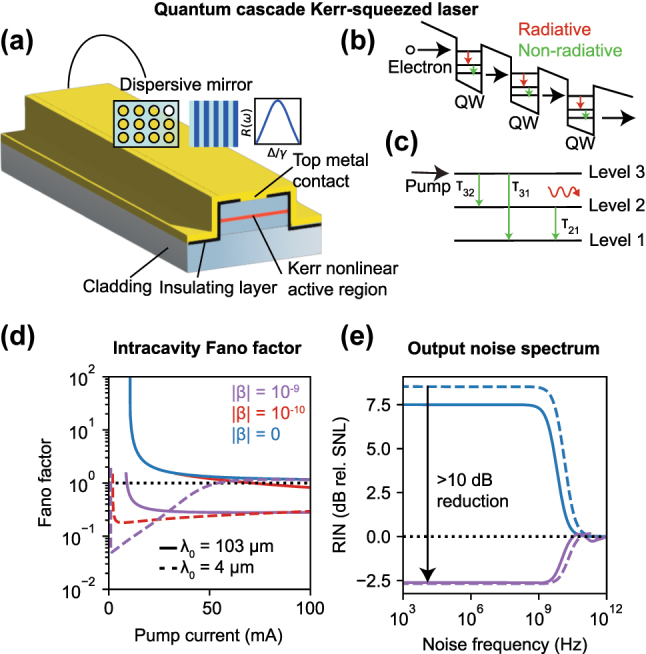
Strongly squeezed mid-IR and terahertz light using QCLs. (a) and (b) Basic dispersive Kerr-squeezed QCL laser architecture with nonlinear dispersive loss. Electrons make subband transitions in a given quantum well and tunnel to the next one. Dispersive outcoupling is provided by a photonic crystal fabricated on an end facet of the QCL. The giant, ultrafast Kerr nonlinearity of the active region due to intersubband transitions is used to generate nonlinear dispersive loss. (c) Three-level system used for rate equation analysis with nonradiative decay timescales from each level indicated. (d) Integrated intracavity Fano factor as a function of pump strength for the low noise branch for three different nonlinear strengths and operating wavelengths in the IR and THz (note that, due to bistability, the low noise branch extends to *I* < *I*_thres_ for strong nonlinearity). (e) Output intensity noise squeezing over GHz-surpassing bandwidths at both mid-IR (dashed) and THz (solid) frequencies. The blue curves correspond to identical loss, but no nonlinearity (*β* = 0). The spectra are plotted for the pump currents corresponding to maximum output squeezing. For these simulations, we use the following system parameters: wavelength *λ*_0_ = 4,103 μm (IR, THz), *τ*_32_ = 2.1 ps, *τ*_31_ = 3.4 ps, *τ*_21_ = 0.5 ps, *m* = 25 gain stages, cavity length *L* = 0.5 mm, and gain coefficient *G*_*N*_ = 2 × 10^5^ s^−1^ [57], [58]. The Fano resonance has FWHM *γ* = 2 × 10^12^ rad/s and is centered at *n*_*c*_ = 10^7^. Intrinsic linear losses are *κ*_0_ = 10^−2^ ⋅ FSR.

A sample design for a QCL with nonlinear dispersive loss is provided in Figure 4a. Here, the intrinsic Kerr nonlinearity of the active region combined with a dispersive mirror on the laser’s output facet generates nonlinear dissipation. To quantify the steady state and noise behavior of this system, we proceed by a Langevin force-based rate equation analysis as before. We use a three-level model for the carrier dynamics (Figure 4b and c), with rate equations describing the evolution of the photon and carrier populations provided in the SI. The nonradiative decay time constants governing transitions between the three carrier levels are given by *τ*_31_, *τ*_32_, *τ*_21_, and linear gain proportional to the difference in population between levels 2 and 3 is assumed.

We calculate intensity noise spectra and integrated Fano factors by Fourier transforming the linearized rate equations (as done above), with details of the calculation provided in the SI. We find that the DC/low-frequency noise is suppressed by a factor ${\left(n_{\text{ss}}\kappa_{n}/\kappa_{\text{ss}}\right)}^{2}$ in the presence of strong nonlinear loss, *n*_ss_ *κ*_*n*_ ≫ *κ*_ss_, 1/*τ*_21_, 1/*τ*_31_, 1/*τ*_32_. We plot the noise behavior for a sample system with Fano mirror outcoupling in Figure 4d and e. In Figure 4d, we consider three different Kerr nonlinear strengths *β* and two different operating wavelengths *λ*_0_ to mimic realistic experimental systems operating in the mid-IR and THz. For comparison, we note that per-photon nonlinear strengths *β* ∼ 10^−10^ were observed nearly two decades ago when QCLs were first used for self-mode-locking [52]. Optimized superlattice design has also been proposed as a method to increase the nonlinear strength by at least another order of magnitude [59]. Our results show that the ultrafast Kerr nonlinearity in QCLs in combination with dispersive loss mechanisms can be harnessed to generate strongly intracavity squeezed states (Figure 4d) and broadband output intensity noise squeezing (Figure 4e), both of which have evaded mid-IR and THz wavelengths.

## 8 Discussion

We briefly describe some of the other experimental platforms for realizing the effects of nonlinear dispersive loss. We have already shown how quantum cascade lasers (QCLs) are promising realizations of semiconductor lasers with nonlinear dispersive loss given their giant, ultrafast Kerr nonlinearities. QCLs emit at IR and THz wavelengths, overlapping with the vibrational modes of many biochemically relevant molecules, making the possibility of developing quantum-enhanced chemosensors or THz data transmitters based on the principles described here tantalizing. The key challenge will be obtaining low background linear losses in these structures, including operating at low temperature and reducing scattering losses from fabrication imperfections, but exciting strides have been taken in this direction to realize ultralow threshold QCLs [60].

Because semiconductor platforms are conducive to integration with on-chip photonic crystal optical elements, many designs have already achieved the dispersive losses considered here and, therefore, could exhibit intensity noise reduction if quality factors and nonlinear strengths are within the tolerances required. For example, previous work has realized “Fano lasers” that exhibit self-pulsing due to the interplay between dispersive loss and carrier nonlinearity [42]. A Fano resonance is created by coupling between a waveguide and nanocavity (point defect) in a photonic crystal slab. Quality factors of *Q*_*γ*_ ∼ 800 have been reported in such Fano lasers, compatible with the photonic crystal resonance widths *γ* considered here. We also note that sharper resonances have been obtained in similar structures using bound states in the continuum [61]. Furthermore, the intrinsic loss due to scattering and material absorption in these devices was *Q*_0_ > 10^2^ *Q*_*γ*_, well within the background losses *κ*_0_ considered here. By integrating a Kerr material in/around the gain region of these lasers, intensity noise reduction by nonlinear dissipation could be observable, strengthened by the low mode volumes in the photonic crystal-based laser cavities.

Photonic crystal surface-emitting lasers (PCSELs) [62], distributed Bragg reflector (DBR) fiber lasers [63], vertical cavity surface-emitting lasers (VCSELs) [64], and DBR diode lasers [65] are other examples of architectures that include sharply frequency-dependent elements that may be used to achieve strong noise condensation. These are established technologies that have now achieved fairly robust fabrication processes, and experimental demonstrations have consistently achieved *Q*_*γ*_ > 10^2^, *κ*_0_/FSR < 10^−1^ for the photonic crystal structure.

We also highlight Kerr microring resonators, which have achieved ultrahigh *Q* factors well in excess of 10^6^ [66], [67], [68], as yet another potential platform to realize the physics described here. It is important to note that the sharpness of the dispersive loss *κ*_*ω*_ in these systems (which can be achieved by coupling a photonic crystal to the resonator) may be restricted because of the requirement that the bandwidth of the resonance exceeds the free spectral range of the resonator (so that the photonic crystal responds “fast enough” to changes in intracavity intensity). However, the low mode volumes in these structures also offer enhanced per-photon Kerr nonlinearity, which may still lead to *F* = *κ*_ss_/*n*_ss_ *κ*_*n*_ ≪ 1.

To introduce appropriate Kerr materials into structures with dispersive loss, the Kerr material should be chosen such that it is passive at the lasing frequency (thereby avoiding resonant effects that would slow its timescale) and approximately index-matched to the gain region to allow significant overlap of the mode profile with the Kerr material (see SI). GaAs-based materials are promising candidates to satisfy both of these criteria. Besides the choice of Kerr material, the geometry of the cavity has the largest influence on the effective nonlinearity, with smaller mode volumes leading to stronger effective nonlinearity. We, therefore, believe quantum well and quantum dot lasers [69], with mode volumes ∼*λ* ^3^, may provide an avenue for strong squeezing via nonlinear dissipation, once the tradeoff between intensity and damage threshold/other parasitic nonlinear effects is optimized.

In this paper, we have shown how sharply frequency-dependent outcoupling and Kerr nonlinearity can endow a laser with nonlinear dissipation, natively supporting intrinsic bistability and self-pulsing capabilities in the mean field, as well as high levels of quantum mechanical intensity noise squeezing both inside and outside the laser cavity. The generality of the mechanism enables squeezing from IR to THz wavelengths, potentially unlocking numerous applications in sensing, computing, and metrology. In contrast to existing bright squeezing techniques, our method is generalizable to different spectral ranges and achieves both broadband output and strong intracavity intensity noise squeezing through elimination of relaxation oscillations. We anticipate that our work will serve as a guide to realize the effects described here in many potential experimental platforms, especially systems employing dispersive loss that could adopt a geometry that maximizes photonic (Kerr) nonlinearity [42].

This work naturally suggests additional possibilities for using nonlinear dissipation to control the output state of lasers and other driven-dissipative systems. Examples of topics for additional investigation include the effect of nonlinear dispersive loss on phase noise and linewidth, the effects of optical feedback on pulsing, bistability, and intensity/phase noise (e.g., in external cavity lasers), and the simultaneous control of self-pulsing and squeezing to generate pulsed squeezing. For example, as we explore in the SI, the sharpness of the dispersive loss *κ*_*ω*_ can significantly impact phase noise and linewidth. We observe linewidth enhancement factors in the O(10) regime for systems like those considered in Section 6.

More generally, nonlinear dissipation is a novel resource to study the interplay between nonlinear dynamics and quantum noise, which is increasingly relevant in integrated platforms that possess strong nonlinearity, dispersive loss, and optical gain. We challenge the widespread assumption that lasers natively produce coherent states, and our work suggests that more exotic quantum states of light can be stabilized in driven-dissipative systems through nonlinear dissipation engineering.

Lasers are ubiquitous sources of coherent light in many real-world applications, and we envision that the use of nonlinear dispersive loss could render them novel tools to control the mean field and noise behavior of light across a wide range of frequencies.

## Supplementary Material

Supplementary Material Details
