# Supplementary material for: Strong broadband intensity noise squeezing from infrared to terahertz frequencies in lasers with nonlinear dissipation
Source: Nanophotonics. 2025 Sep 19;14(20):3243–54. doi: 10.1515/nanoph-2025-0259 (PMC12588569; doi:10.1515/nanoph-2025-0259)
Supplement: Supplementary file 1 — Supplementary Material Details [file j_nanoph-2025-0259_suppl_001.pdf]

Supplementary Information for:
Strong broadband intensity noise squeezing from infrared to terahertz frequencies in lasers with nonlinear dissipation

Sahil Pontula[1,2,3,*,†], Jamison Sloan[4,†], Nicholas Rivera[5,6], and Marin Soljačić[1,3]

[1]*Department of Physics, MIT,*
Cambridge, MA 02139, USA.

[2]*Department of Electrical Engineering and Computer Science,*
MIT, Cambridge, MA 02139, USA.

[3]*Research Laboratory of Electronics,*
MIT, Cambridge, MA 02139, USA.

[4] *E. L. Ginzton Laboratory,*
Stanford University,
Stanford, CA 94305, USA

[5] *School of Applied and Engineering Physics,*
Cornell University, Ithaca,
New York 14853, USA

[6]*Department of Physics,*
Harvard University,
Cambridge, MA 02138, USA.

∗ *Contact: spontula@mit.edu.*

† *Denotes equal contribution.*

Abstract

In this Supplementary Information (S.I.), we present derivations of results reported in the main text, further details about the systems considered, and supplemental figures. Included in these discussions are closer examinations of mean field self-pulsing and bistability, the effect of carrier-dependent shifts to the refractive index on quantum noise, mean field and noise behavior for a distributed feedback-based nonlinear dissipation profile, linewidth and phase noise, and a derivation of output noise in the presence of nonlinearity and dispersive loss.

Contents

1. Heisenberg-Langevin equations of motion for a conventional semiconductor laser 4

2. Carrier equation of motion under electrical and optical excitation 7

3. Estimation of per-photon Kerr nonlinearity β 7

4. Mean field dynamics: bistability and self-pulsing 8

A. Bistability due to Kerr nonlinearity 8

B. Onset and cessation of self-pulsing 9

C. Pulse characteristics in self-pulsing regime 9

5. Intensity noise 11

A. Langevin force correlators 11

B. Analytic intensity noise spectra and Fano factor expressions 12

C. Noise reduction using two photon absorption (TPA) 15

D. Noise reduction using nonlinear distributed feedback-based loss 16

1. Comparison of Fano mirror and DBR loss profiles 18

6. Linewidth and phase noise 19

7. Output photon noise in lasers with nonlinear dispersive loss 21

8. Intensity noise in QCLs with nonlinear dispersive loss 24

9. Nonlinear dispersive loss with carrier and Kerr nonlinearities 26

10. Quantum phase space distribution in systems with nonlinear dissipation 27

References 28

1. HEISENBERG-LANGEVIN EQUATIONS OF MOTION FOR A CONVENTIONAL SEMICONDUCTOR LASER

In line with the main text, for clarity of notation, we will use $\hat{X}$ to denote an arbitrary quantum mechanical operator, $X \equiv \langle \hat{X} \rangle$ to denote the mean field value of this operator, and X_{ss} to denote its steady state mean field value.

The Hamiltonian of a simple two-band semiconductor can be writen as [S1]

$$\hat{H}_{\text{SC}} = \sum_q (\epsilon_g^{(0)} + \epsilon_{e,q}) \hat{c}_q^\dagger \hat{c}_q + \sum_q \epsilon_{h,q} \hat{h}_q^\dagger \hat{h}_q + \hat{V}_{\text{int}}. \tag{S1}$$

Here, $\hat{c}_q$ and $\hat{h}_q$ are the fermionic annihilation operators for conduction-band electrons and valence-band holes at momentum q. They satisfy the fermionic commutation relations $\{\hat{c}_q, \hat{c}_{q'}^\dagger\} = \delta_{qq'}$, and likewise for $\hat{h}_q$. Additionally, $\epsilon_g^{(0)}$ is the unrenormalized bandgap energy which separates the two bands at zero momentum. Also note that the sums $\sum_q$ are intended to note a sum over all electron states q, including momentum, spin, and anything else that might be relevant. Finally, $\hat{V}_{\text{int}}$ represent interactions (collisions between electrons, interactions of the electron with the lattice, etc.). We will not need to consider the effects of this term, but its presence will lead to effects such as collision-induced equilibration of carriers within a band, relaxation of carriers from the upper band to the lower band, etc. Interactions can also lead to some shifts in the gain spectrum induced by carrier screening and band-gap renormalization.

Now, we would like to introduce a single mode Kerr nonlinear cavity which has frequency ω_0 and annihilation operator $\hat{a}$, so that the Hamiltonian of the cavity is $\hat{H}_{\text{cavity}} = \omega_0 \hat{a}^\dagger \hat{a} \left(1 + \beta \hat{a}^\dagger \hat{a}\right)$ with β the per-photon Kerr nonlinearity. In order to describe lasing, the cavity should interact with the semiconductor gain medium through its dipole moment. We can define analogs of the atomic raising/lowering operators $\hat{\sigma}_\pm$ for each electron label q as $\hat{\sigma}_q \equiv \hat{c}_q \hat{h}_q$. Then, in the rotating wave approximation (which assumes the light-matter coupling between the light and semiconductor is weak), the interaction between cavity and semiconductor is

$$\hat{H}_{\text{int}} = \sum_q \left(g_q \hat{a} \hat{\sigma}_q^\dagger + g_q^* \hat{\sigma}_q \hat{a}^\dagger \right). \tag{S2}$$

Then the Hamiltonian of the full laser is the sum of the contributions $\hat{H} = \hat{H}_{\text{SC}} + \hat{H}_{\text{cavity}} + \hat{H}_{\text{int}}$. Our goal then is to write equations of motion for quantities of interest, and then solve these equations for steady state, transient, and noise properties of the laser. To do so, we will now write Langevin

equations of motion for the semiconductor laser. This amounts to computing the Heisenberg equations of motion for the operators of interest, adding the relevant pumping and damping terms, and finally computing the correlations between the Langevin forces which results to describe noise behavior.

For the polarization operator, we find

$$\dot{\hat{\sigma}}_q = -i\omega_q \hat{\sigma}_q - \gamma_\perp \hat{\sigma}_q + i g_q \hat{a}(\hat{n}_{e,q} + \hat{n}_{h,q} - 1) + \hat{f}_q, \tag{S3}$$

where ω_q is the energy difference between the valence and conduction bands for state q. We see that σ_q oscillates in the same way that σ_i does for an atomic gain medium. Additionally, we see that the quantity in parentheses (which we shall define as $\hat{d}_q$) in the second term acts like the inversion in an atomic gain medium. Specifically, the occupation operators for the electrons and holes can both takes values between 0 and 1. For a completely unexcited state ($n_e = n_h = 0$), the grouped quantity is $d_q = -1$. For a completely excited state ($n_e = n_h = 1$) we have $d_q = 1$. Thus d_q can be thought of as the population inversion for each electron state q.

For the cavity photon annihilation operator,

$$\begin{aligned} \dot{\hat{a}} &= -i\omega_0 \left(1 + \beta \hat{a}^\dagger \hat{a}\right) \hat{a} - \frac{\kappa}{2}\hat{a} - i \sum_q g_q^* \hat{\sigma}_q + \hat{f}_a \\ &= -i\omega_0 \hat{a} \left(1 + \beta \hat{a}^\dagger \hat{a}\right) a - \frac{\kappa}{2}\hat{a} + \frac{\hat{a}}{\gamma_\perp} \sum_q |g_q|^2 \mathcal{D}_q \hat{d}_q + \hat{f}_a, \end{aligned} \tag{S4}$$

where κ is the cavity number/energy damping rate, $\mathcal{D}_q \equiv \frac{\gamma_\perp}{i(\omega-\omega_q)+\gamma_\perp}$ and $\hat{f}_a$ is the Langevin force for the annihilation operator. In the second line, we adiabatically eliminated the polarization. Note that β represents the per-photon Kerr nonlinear strength.

Lastly, for the electron occupation operator,

$$\dot{\hat{n}}_{e,q} = \Lambda_{e,q}(1 - \hat{n}_{e,q}) - B_q \hat{n}_{e,q}\hat{n}_{h,q} - \gamma_\parallel \hat{n}_{e,q} - \gamma_e(\hat{n}_{e,q} - (\hat{n}_{e,q})_0) + i g_q^* \hat{a}^\dagger \hat{\sigma}_q - i g_q \hat{\sigma}_q^\dagger \hat{a} + \hat{F}_{e,q}. \tag{S5}$$

In order from left to right, the terms are

- **Population pumping.** This is the pump rate due to carrier injection. When summing over this term, we get the actual pump rate I at which free carriers are injected.
- **Loss due to spontaneous emission.** Excited carriers can be lost due to spontaneous emission. Since different q can have different energy splittings, one of these spontaneous emis-

sion events will not necessarily be into the laser mode of interest. The coefficient B_q is the rate for a particular momentum state q.

- **Nonradiative decay of population.** This term represents the rate at which exited carriers become unexcited in a manner which is proportional to the population (e.g., due to phonon emission).
- **Carrier-carrier relaxation.** This term represents relaxation to the equilibrium value $(n_{e,q})_0$ within a band. The fact that γ_e tends to be very large compared to other relaxation rates allows one to make the so-called "quasiequilibrium" approximation in which each band acquires a Fermi-Dirac distribution. Moreover, because this term only redistributes carriers to different q within the same band, it does not have an effect on the total inverted population. Thus, when summing this term over electron states, it vanishes.
- **Population depletion by stimulated emission into cavity mode.** This is the only term that can be derived from the Hamiltonian written above. This is the term that causes the population of excited states to deplete when stimulated emission occurs.

Now, we identify in the mean field

$$G(N)(1 - i\alpha_L) \equiv \frac{2}{\gamma_\perp} \sum_q |g_q|^2 d_q \mathcal{D}_q \tag{S6}$$

$$\Gamma(N) \equiv \gamma_{\|} N + \frac{1}{V} \sum_q B_q n_{e,q} n_{h,q} \tag{S7}$$

$$N \equiv \sum_q n_{e,q}. \tag{S8}$$

where the linewidth enhancement factor $\alpha_L \equiv \frac{d\chi_r/dN}{d\chi_i/dN}$, with $\chi = \chi_r + i\chi_i$ the susceptibility of the active material [S2]. We can now identify the resonance frequency using $a \equiv \alpha e^{i\phi}$ and $\dot{\phi} = \frac{1}{2i}\frac{d}{dt}\ln\left(\frac{a}{a^*}\right) = \frac{1}{2i}\left(\frac{\dot{a}}{a} - \frac{\dot{a}^*}{a^*}\right)$, showing that

$$\omega_0 \to \omega_0 \left(1 + \beta n - \frac{\alpha_L}{2\omega_0} G(N)\right), \tag{S9}$$

so that the "carrier nonlinearity" is identified as $\sigma \equiv -\alpha_L G_N/2\omega_0$. With these substitutions and neglecting the effects of spontaneous emission, the Heisenberg-Langevin equations in the main text are obtained.

Here, we neglected any frequency-dependent phase shifts imparted by the Fano mirror. These can be rigorously incorporated into the Heisenberg-Langevin equations using coupled mode theory, as we do below in Sec. 7. The result is a phase shift $\tan^{-1}(\Delta(\omega_R)/\gamma)$, where $\Delta(\omega_R)$ represents the detuning from the Fano resonance and γ the width of the Fano resonance. We assume the second cavity mirror (back reflector) is broadband and imparts no phase shift. The effect of including the Fano mirror's phase shift is to make the resonance frequency no longer analytically solvable given n, N using Eq. S9. Instead, it must be solved numerically. However, we find that the effect of this dispersive phase shift is negligible over the detunings we consider: sweeping across the Fano resonance gives a deviation from the prediction of Eq. S9 of at most 0.02γ, likely from the broad width we assume for the Fano resonance under the adiabatic approximation.

2. CARRIER EQUATION OF MOTION UNDER ELECTRICAL AND OPTICAL EXCITATION

In the main text, electrical pumping is assumed, in which case the mean field carrier equation of motion derived from the Heisenberg-Langevin formalism reads

$$\dot{N} = \eta I_p - \gamma_{\|} N - nG(N), \tag{S10}$$

with $I = \eta I_p$ the pump current, η the pump efficiency, and N the carrier number. In the case of free carrier excitation due to optical pumping, the mean field carrier equation of motion derived from the Heisenberg-Langevin formalism reads [S3]

$$\dot{N} = \frac{\eta P_p}{\hbar\omega_p V_p} - \gamma_{\|} N - nG(N), \tag{S11}$$

where η is the pump efficiency, P_p the pump power, $\hbar\omega_p$ the energy of a pump photon, and V_p the pump volume. Assuming a pump volume V_p on the order of the active region volume and excitation by a near-IR source (around 800 nm), typical pump powers are on the order of tens of mW for examples considered in the main text with pump currents on the order of tens of mA.

3. ESTIMATION OF PER-PHOTON KERR NONLINEARITY β

We briefly describe how the per-photon Kerr nonlinearity β can be estimated. Previous work has derived the per-photon Kerr nonlinearity from a quantum mechanical Hamiltonian approach [S4]:

$$\beta = \frac{3\hbar\omega_0}{8\epsilon_0^2} \int \chi^{(3)}(\mathbf{r}) |\mathbf{u}(\mathbf{r})|^4 d^3\mathbf{r}, \tag{S12}$$

where the electric field profile is normalized as $\int |\mathbf{u}(\mathbf{r})|^2 \epsilon_r(\mathbf{r}) d^3\mathbf{r} = 1$. To get an estimate of achievable β, we consider a buried heterostructure laser with GaAs gain and $Al_{0.34}Ga_{0.66}As$ cladding. The active region has dimensions 0.1 μm $\times$ 5 μm $\times$ 1 mm, the lasing frequency is near the bandgap of GaAs, $\omega_0 = 2.16 \times 10^{15}$ rad/s, and the refractive indices of GaAs and $Al_{0.34}Ga_{0.66}As$ are $n_{\text{core}} = 3.6051$ and $n_{\text{clad}} = 3.3734$ respectively. We take $n_2 \approx -10^{-16}$ m^2/W for $Al_{0.34}Ga_{0.66}As$ [S5]. We solve Maxwell's equations in the core and cladding using a slab waveguide model in which the transverse field profile reads

$$\begin{aligned} E_y(y < d/2) &\sim \cos(\kappa y) \\ E_y(y > d/2) &\sim \cos(\kappa d/2) e^{-\gamma(|y| - d/2)}, \end{aligned} \tag{S13}$$

where $d = 10$ μm is the thickness of the GaAs active region and

$$\kappa = k_0 \sqrt{n_{\text{core}}^2 - n_e^2} \tag{S14}$$

$$\gamma = k_0 \sqrt{n_e^2 - n_{\text{clad}}^2}, \tag{S15}$$

with $k_0 = \omega_0 / c$. The effective index n_e is solved through the transcendental equation

$$\gamma(n_e) = \kappa(n_e) \tan(\kappa(n_e)/2). \tag{S16}$$

From the resulting mode profile, we obtain a confinement factor $\Gamma \approx 0.3$ and per-photon Kerr nonlinearity $\beta \approx -6 \times 10^{-10}$. The mode has approximate dimensions 50 μm $\times$ 50 μm in this structure.

4. MEAN FIELD DYNAMICS: BISTABILITY AND SELF-PULSING

A. Bistability due to Kerr nonlinearity

Here, we quantify the bistability boundaries that arise when intensity-dependent loss is present. As shown in Fig. 2, this bistability correlates with the phenomenon of self-pulsing and demarcates an unstable region in the S-curve for the laser. Its boundaries can be found by noting that, in the steady state,

$$\begin{aligned} I(n) &= \gamma_{\|} N(n) + n G_N \left(N(n) - N_{\text{trans}} \right) \\ N(n) &= \frac{\kappa(n)}{G_N} + N_{\text{trans}}. \end{aligned} \tag{S17}$$

The bistability boundaries (in pump I) are those values $I(n)$ for which $dI/dn = 0$, for which we require

$$\frac{dI}{dn} = 0 \implies \kappa_n \left(\frac{\gamma_{\parallel}}{G_N} + n \right) + \kappa(n) = 0. \tag{S18}$$

One can see, for example, that in the absence of intrinsic loss, n_c, the point of zero loss, satisfies this condition, since $\kappa_n(n_c) = \kappa(n_c) = 0$.

B. Onset and cessation of self-pulsing

Self-pulsations begin when relaxation oscillations become undamped, $\Gamma_1 < 0$ and $\Omega_R^2 > 0$ (in the initial steady state solution). They do not, however, persist throughout the entire region where $\Gamma_1 < 0$, as shown in Fig. 2 of the main text. When the laser begins at the left edge of bistability in the S-curve (as in Fig. 2b) at steady state, the pulsations are transient and eventually collapse to the steady state solution at the center of the Fano resonance with minimum loss at photon number n_c (this is the leftmost point of bistability). Note that the laser began at the second photon number n^* (low intensity branch) that corresponds to the same pump power as photon number n_c. Eventually, we sweep through initial steady state photon numbers within the region of instability (still within bistable operation) that is not normally accessible by pumping directly from threshold. Within this region, the laser switches to the upper or lower stable branch of the S-curve (depending on whether a transient increase or decrease in the photon number occurs). The switching timescale is roughly set by the inverse relaxation oscillation damping rate $1/\Gamma_1$. When the right edge of bistability is finally crossed, the laser enters the region with $\kappa_n > 0$, characterized by heavily damped relaxation oscillations and intensity noise squeezing. When pumping from threshold, the laser jumps from the low intensity to high intensity branch at the right bistable edge.

C. Pulse characteristics in self-pulsing regime

The self-pulsations demonstrate an interesting behavior in pulse shape, as shown in Fig. S1. The initial sharp rise in the pulse profile is due to the undamping of relaxation oscillations, and its timescale is thus set by $1/|\Gamma_1|_{\max} \approx 1/|n\kappa_n|_{\max}$ ($\mathcal{O}(1)$ ps in our simulations). The same timescale characterizes the final drop in pulse power. In between these two features, two further timescales are at play. The decay after peak pulse power is initially very fast ($\mathcal{O}(1)$ ps) due to the

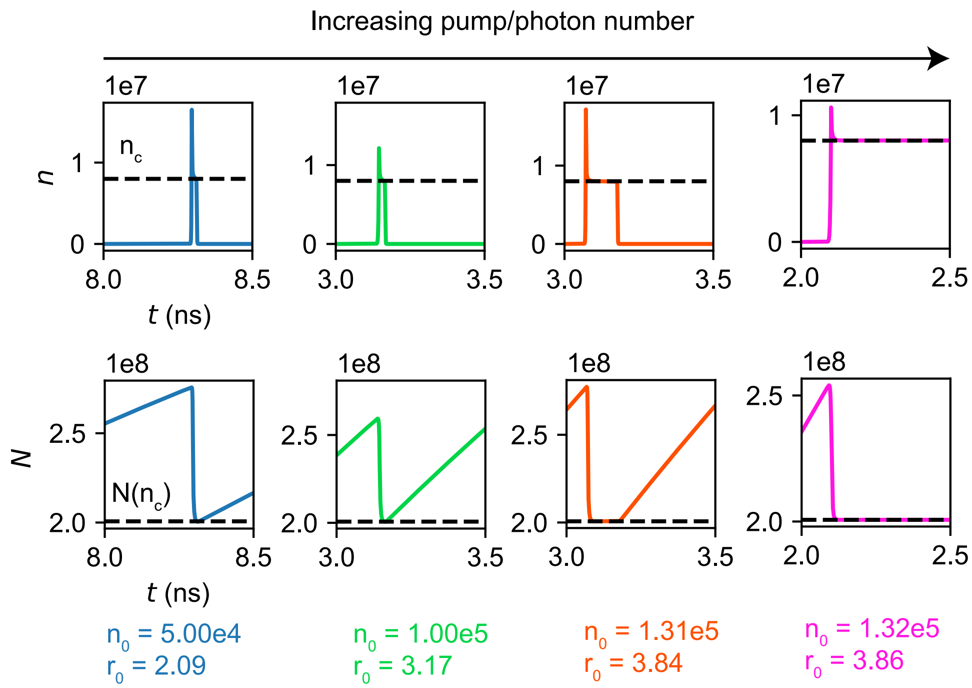

FIG. S1: **Evolution of the pulse profile for carrier density and photon number from the self-pulsing to collapsed pulse regimes.** As the initial photon number n_0 approaches the left bistable edge, the pulse plateaus for longer at the center of the Fano resonance. Thus, the effective width of the pulse is dynamic within the regime over which self-pulsing occurs, depending on the initial state's proximity to the left bistable edge. Once the left bistable edge is crossed, the pulse collapses to a CW solution at higher photon number than the initial state. Here, r_0 denotes the initial pumping rate relative to threshold.

strong damping of relaxation oscillations in the $\kappa_n > 0$ region. The decay slows as the photon number approaches n_c, governed by Γ_1 evaluated at $n \approx n_c$. The final feature also sets the longest timescale for the pulse. It is a plateau near $n \approx n_c$ that emerges from "quasi" steady state conditions. The carrier density can be calculated by solving the carrier equation of motion in the steady state as

$$N \approx \frac{I_0 + n_c G_N N_{\text{trans}}}{\gamma_{||} + n_c G_N}, \tag{S19}$$

where I_0 denotes the (fixed) pumping rate. Notice here that for $n < n^*$, $N < N(n_c)$, so that $G(N) < \kappa(n)$ as the photon number drops below n_c and approaches the point where the pulsing continues ($\Gamma_1 < 0$). The timescale for the plateau is then given by $\tau_p = 1/|G(N) - \kappa(n_c)|$. Notice that $N \to N_c, \tau_p \to \infty$ as the initial steady state photon number $n_0 \to n^*$. When $n_0 = n^*$, the pulsations are transient and the laser approaches a steady state at $n_0 = n_c$, as shown in Fig. S1.

For $n_0 \approx n^*$, the plateau changes based on the initial steady state (i.e. pumping rate) and can approach timescales of tens to hundreds of ps.

The peak pulse power is more difficult to predict, depending on the initial fluctuation from steady state. However, it must occur at $n > n_c$ to saturate the pulse and begin its decline.

The pulse repetition rate is set by the carrier density recovery timescale when the pulse is off. During this time, the photon number $n \approx 0$, so that the mean field dynamics of carrier density are given by

$$N(t) = \left(N_{\min} - \frac{I}{\gamma_\parallel}\right) e^{-\gamma_\parallel t} + \frac{I}{\gamma_\parallel}, \tag{S20}$$

where I denotes the pump current and $N_{\min}$ the minimum carrier density. If $\Delta N = N_{\max} - N_{\min}$ is the difference in carrier density at the pulse maximum and minimum, the period between pulses is given roughly by

$$T_{\text{rep}} \approx \frac{1}{\gamma_\parallel} \ln\left(\frac{I/\gamma_\parallel - N_{\min}}{I/\gamma_\parallel - N_{\max}}\right). \tag{S21}$$

For the system parameters in the main text, $T_{\text{rep}} \sim 0.1 - 10$ ns (100 MHz-10 GHz repetition rate).

5. INTENSITY NOISE

A. Langevin force correlators

In this section, we derive the photon number correlator in the presence of two-photon absorption (TPA). We begin with the equation of motion for photon number probabilities in the presence of TPA only, $\dot{p}_n = -\frac{\alpha_{\text{TPA}}}{2} n(n-1)p_n + \frac{\alpha_{\text{TPA}}}{2}(n+1)(n+2)p_{n+2}$, where p_n denotes the probability of having n photons inside the laser cavity. Thus,

$$\begin{aligned}
\dot{\langle \hat{n} \rangle} &= \sum_j j\dot{p}_j \\
&= -2\alpha_{\text{TPA}} \sum_j j(j-1)p_j \\
&= -\alpha_{\text{TPA}}[\langle \hat{n}^2 \rangle - \langle \hat{n} \rangle].
\end{aligned} \tag{S22}$$

The RHS reduces to $-\alpha_{\text{TPA}}\langle \hat{n} \rangle^2$ assuming mean field theory, $\Delta n \ll \langle \hat{n} \rangle$, recovering the mean field equation of motion $\dot{n} = -\alpha_{\text{TPA}} n^2$. Using the generalized Einstein relation, the correlator is

$\langle 2\hat{D}_{nn}\rangle = \frac{d}{dt}\langle \hat{n}^2\rangle - 2\langle \hat{n}\hat{D}_n\rangle$, where we express $\dot{\hat{n}} = \hat{D}_n + \hat{F}_n$, with $\hat{D}_n$ a diffusion term and $\hat{F}_n$ a Langevin force. Thus

$$\begin{aligned}\langle 2\hat{D}_{nn}\rangle &= \left(\sum_j j^2 \dot{p}_j\right) + 2\alpha_{\mathrm{TPA}}\langle \hat{n}^3 - \hat{n}^2\rangle \\ &= -\alpha_{\mathrm{TPA}}\langle \hat{n}(\hat{n}-1)^2\rangle + 2\alpha_{\mathrm{TPA}}\langle \hat{n}^2(\hat{n}-1)\rangle \\ &\approx 2\alpha_{\mathrm{TPA}}\langle \hat{n}\rangle^2,\end{aligned} \tag{S23}$$

again assuming mean field theory. Allowing for one-photon gain and loss, $\langle 2\hat{D}_{nn}\rangle = 2\kappa n + \alpha_{\mathrm{TPA}} n^2$. The other nonzero diffusion coefficients are $\langle \hat{F}_N^\dagger \hat{F}_N\rangle = \langle 2\hat{D}_{NN}\rangle = \epsilon I + R_{sp}n + \gamma_{\|}N$, $\langle \hat{F}_N^\dagger \hat{F}_n\rangle = \langle 2\hat{D}_{Nn}\rangle = -Rn$, $\langle \hat{F}_\phi^\dagger \hat{F}_\phi\rangle = \langle 2\hat{D}_{\phi\phi}\rangle = R_{sp}/2n$ where $R_{sp} \approx G(n, N)$ denotes the rate of spontaneous emission into the cavity mode, $R_{abs} \approx 0$ denotes the rate of absorption (negligible above threshold), $R = R_{sp} + R_{abs}$, and $\epsilon = 0$ (1) for quiet (noisy) pumping. These correlators can be derived by computing the Einstein diffusion coefficients [S1] and give rise to nonzero fluctuations in n, N about their steady state values. For intracavity noise calculations in the main text, pump noise is always included. Output noise calculations are performed for both noisy and quiet pumping schemes.

B. Analytic intensity noise spectra and Fano factor expressions

In this section, we provide a linearization of the semiconductor laser rate equations in the presence of various nonlinearities and calculate relative intensity noise using this formalism. Linearizing mean field Eq. 3 in the main text and augmenting with Langevin force terms $\hat{F}_{n,N,\phi}$ for the equations of motion $\dot{n}, \dot{N}, \dot{\phi}$,

$$\begin{aligned}\delta\dot{\hat{n}} &= n_{\mathrm{ss}}\frac{\partial G}{\partial n}\delta\hat{n} + n_{\mathrm{ss}}\frac{\partial G}{\partial N}\delta\hat{N} - n_{\mathrm{ss}}\frac{\partial \kappa}{\partial \omega}\delta\hat{\omega} + \hat{F}_n \\ \delta\dot{\hat{N}} &= \left(\frac{\partial I}{\partial n} - n_{\mathrm{ss}}\frac{\partial G}{\partial n} - G_{\mathrm{ss}}\right)\delta\hat{n} - \left(\gamma_{\|} + n_{\mathrm{ss}}\frac{\partial G}{\partial N}\right)\delta\hat{N} + \hat{F}_N,\end{aligned} \tag{S24}$$

where all partial derivatives are evaluated at the steady state values $n_{\mathrm{ss}}, N_{\mathrm{ss}}, \omega_{\mathrm{ss}}$. Note that $G_{\mathrm{ss}} = \kappa_{\mathrm{ss}}$ in steady state, so we will use these two interchangeably. Note also that $\partial G/\partial n$ and $\partial I/\partial n$ can be nonzero due to gain saturation and carrier generation by TPA. As in the main text, we will neglect these effects here, but they can be rigorously incorporated into our treatment. Using

$\delta\hat{\omega} = \delta\dot{\hat{\phi}} = \frac{\partial\omega}{\partial N}\delta\hat{N} + \frac{\partial\omega}{\partial n}\delta\hat{n} + \hat{F}_\phi$ [S6], we thus have

$$\begin{aligned} \delta\dot{\hat{n}} &= (-\kappa_n n_{\rm ss})\delta\hat{n} + n_{\rm ss}\left(G_N - \kappa_N\right)\delta\hat{N} + \hat{F}_n - n_{\rm ss}\kappa_\omega \hat{F}_\phi \\ \delta\dot{\hat{N}} &= -G_{\rm ss}\delta\hat{n} - \left(G_N n_{\rm ss} + \gamma_\parallel\right)\delta\hat{N} + \hat{F}_N, \end{aligned} \tag{S25}$$

where $\kappa_\omega \equiv \partial\kappa/\partial\omega$ and $\kappa_n \equiv \partial\kappa/\partial n = \kappa_\omega(\partial\omega/\partial n)$ and similarly for κ_N.

For simplicity of notation, we will introduce $a = n_{\rm ss}G_N + \gamma_\parallel, b = n_{\rm ss}\left(G_N - \kappa_N\right), c = G_{\rm ss}, d = n_{\rm ss}\kappa_n, \Gamma_1 = a + d, \Omega_R^2 = ad + bc$. Note that Ω_R^2 denotes the approximate relaxation oscillation frequency and Γ_1 the decay of relaxation oscillations. Fourier transforming the linearized rate equations,

$$\begin{bmatrix} -i\Omega + d & -b \\ c & -i\Omega + a \end{bmatrix} \begin{bmatrix} \delta\hat{n}(\Omega) \\ \delta\hat{N}(\Omega) \end{bmatrix} = \begin{bmatrix} \hat{F}_n - n_{\rm ss}\kappa_\omega\hat{F}_\phi \\ \hat{F}_N \end{bmatrix}, \tag{S26}$$

yielding

$$\begin{bmatrix} \delta\hat{n}(\Omega) \\ \delta\hat{N}(\Omega) \end{bmatrix} = \frac{1}{-\Omega^2 + (ad + bc) - i\Omega(a + d)} \begin{bmatrix} (-i\Omega + a)\left(\hat{F}_n - n_{\rm ss}\kappa_\omega\hat{F}_\phi\right) + b\hat{F}_N \\ -c\left(\hat{F}_n - n_{\rm ss}\kappa_\omega\hat{F}_\phi\right) + (-i\Omega + d)\hat{F}_N. \end{bmatrix} \tag{S27}$$

The intensity noise spectrum is then

$$\langle\delta\hat{n}^\dagger(\Omega)\delta\hat{n}(\Omega)\rangle = \frac{(\Omega^2 + a^2)[\langle 2\hat{D}_{nn}\rangle + n_{\rm ss}^2\kappa_\omega^2\langle 2\hat{D}_{\phi\phi}\rangle] + b^2\langle 2\hat{D}_{NN}\rangle + 2ab\langle 2\hat{D}_{Nn}\rangle}{(\Omega^2 - \Omega_R^2)^2 + \Omega^2\Gamma_1^2}. \tag{S28}$$

As a side note, ignoring the effect of Kerr nonlinearity but including dispersive loss and the associated amplitude-phase coupling, we see that RIN can be reduced by a factor $(1 + \kappa_\omega^2)/(1 - \alpha_L\kappa_\omega/2)^2 \to 1/(1 + \alpha_L^2)$ if the slope κ_ω is chosen appropriately, in agreement with earlier work on amplitude-phase decorrelation (where intensity noise is reduced somewhat at the expense of an increase in phase noise) [S7]. However, this method leads to frequency selective squeezing, as opposed to the type of broadband squeezing we consider here.

We compute the Fano factor from Eq. S28 using the integrals

$$I_1 = \int_0^\infty \frac{1}{(\omega^2 - x^2)^2 + y^2} d\omega = \frac{\pi}{4y} \frac{\sqrt{2x^2 + 2\sqrt{x^4 + y^2}}}{\sqrt{x^4 + y^2}}$$

$$I_2 = \int_0^\infty \frac{\omega^2}{(\omega^2 - x^2)^2 + y^2} d\omega = \frac{\pi}{4} \frac{\sqrt{-2x^2 + 2\sqrt{x^4 + y^2}}}{\sqrt{x^4 + y^2}} + x^2 I_1,$$

where $x, y \in \mathbb{R}$. With $x^2 = \Omega_R^2 - \frac{\Gamma_1^2}{2}, y^2 = \Gamma_1^2 \left(\Omega_R^2 - \frac{\Gamma_1^2}{4}\right)$, we have $I_1 = \frac{\pi}{2\Gamma_1 \Omega_R^2}, I_2 = \frac{\pi}{2\Gamma_1}$. Thus, the Fano factor reads

$$F = \frac{1}{2n\Gamma_1\Omega_R^2} \left([\langle 2\hat{D}_{nn} \rangle + n_{\text{ss}}^2 \kappa_\phi^2 \langle 2\hat{D}_{\phi\phi} \rangle](\Omega_R^2 + a^2) + \langle 2\hat{D}_{Nn} \rangle ab + \langle 2\hat{D}_{NN} \rangle b^2 \right) \tag{S29}$$

We now consider limiting expressions for F in various limiting cases:

1. For weak Kerr and carrier nonlinearities, $\kappa_n, \kappa_N \to 0$, we have $F \to 1 + \kappa_{\text{ss}}/(n_{\text{ss}} G_N)$ when pumping far above threshold, recovering linear behavior. When n_{ss} becomes large far about threshold, the Fano factor approaches 1, resulting in Poissonian (coherent) statistics.

2. For strong Kerr nonlinearity but weak carrier nonlinearity, $n_{\text{ss}}\kappa_n \gg \kappa_0, n_{\text{ss}}|\kappa_N|, \gamma_\parallel, G_N$, the Fano factor $F \to \kappa_{\text{ss}}/(n_{\text{ss}}\kappa_n)$ for large n_{ss}, resulting in squeezing when $n_{\text{ss}}\kappa_n > \kappa_{\text{ss}}$.

3. For strong carrier nonlinearity but weak Kerr nonlinearity, $n_{\text{ss}}|\kappa_N| \gg \kappa_0, n_{\text{ss}}|\kappa_n|, \gamma_\parallel$, we have $F \to \kappa_{\text{ss}}/(n_{\text{ss}} G_N) + G_N/|G_N - \kappa_N| \to G_N/|G_N - \kappa_N|$ for large n_{ss}. The carrier nonlinearity reduces dependence of the rate of change of intensity fluctuations on carrier fluctuations ($G_N \to G_N - \kappa_N$), lowering the relaxation oscillation frequency Ω_R^2 while leaving the damping of these oscillations unchanged. This can amplify low-frequency intensity noise slightly.

4. For simultaneously strong Kerr and carrier nonlinearities, $n_{\text{ss}}|\kappa_{n,N}| \gg \kappa_0, \gamma_\parallel$,

$$F \to \frac{\kappa_{\text{ss}}}{n_{\text{ss}}|G_N + \kappa_n|} \left(1 + \frac{n_{\text{ss}} G_N^2}{n_{\text{ss}}\kappa_n G_N + |G_N - \kappa_N|\kappa_{\text{ss}}} \right). \tag{S30}$$

Roughly, this expression can be broken into Kerr nonlinearity (first term) and carrier nonlinearity (second term) contributions. The former describes squeezing via increased Ω_R^2 and damping of relaxation oscillations due to "sharp" intensity-dependent loss, while the latter reduces intensity noise-carrier noise coupling and thus Ω_R^2. Kerr and carrier nonlinearities

may therefore have competing effects, leading to interesting steady state and noise fluctuation behavior.

C. Noise reduction using two photon absorption (TPA)

Two photon absorption (TPA), though not a dispersive loss, is weakly nonlinear in photon number and thus may be expected to permit some squeezing in intensity noise. When TPA is present, for large $n_{\rm ss}$,

$$F \to \frac{3\kappa_{\rm ss}}{2n_{\rm ss}(G_N + \alpha_{\rm TPA})}\left(1 + \frac{G_N}{\alpha_{\rm TPA} + \kappa_{\rm ss}/n_{\rm ss}}\right), \tag{S31}$$

where $\alpha_{\rm TPA} = \kappa_n$. The minimum achievable Fano factor is 3/4, obtained when $\kappa_0/n_{\rm ss} \ll \alpha_{\rm TPA} \ll G_N$ (here κ_0 denotes linear background loss). To obtain the TPA coefficient $\alpha_{\rm TPA}$, we use the relationship between intensity I and photon number $I \sim n\hbar\omega c/V$, so that $\alpha_{\rm TPA} \sim 2\hbar\omega c L \beta_{\rm TPA} \cdot {\rm FSR}/V$, where L, V respectively denote the length and volume of the cavity. For a cavity field oscillating at $\omega \sim 10^{15}$ Hz for GaAs at 1064 nm ($\beta_{\rm TPA} = 260$ m/TW), we find $\alpha_{\rm TPA} \sim 10^{-8} \cdot {\rm FSR}$ for $L \approx 1$ mm, $V \approx 10^{-16}$ m^3. For typical intracavity photon numbers, the TPA contribution to the loss is then $10^{-2} \cdot {\rm FSR}$, a weak nonlinear background loss that is neglected for the examples in the main text where the primary nonlinear dispersive loss is much stronger.

As shown in Fig. S2a, TPA creates a sublinear S-curve that arises from the monotonic dependence of $\kappa(n)$ on n. Fig. S2b demonstrates how TPA induces broadband intensity noise squeezing, resulting in a weak suppression of Fano factor (integrated over all noise frequencies) in Fig. S2c. Linear loss asymptotes to unit Fano factor for large pump powers, while TPA can result in minor noise condensation (though this effect can be washed out if TPA is too strong or too weak, in violation of $\kappa_0/n_{\rm ss} \ll \alpha_{\rm TPA} \ll G_N$). The source of Fano factor reduction for higher pump currents is slightly different for both loss profiles. For linear loss, it occurs because steady state n increases linearly with pump current while the fluctuations $(\Delta n)^2$ have a sublinear dependence on pump current. In contrast, for TPA, the photon number n is clamped at high pump current and the photon number distribution is squeezed slightly due to the nonlinear loss $\kappa(n)$.

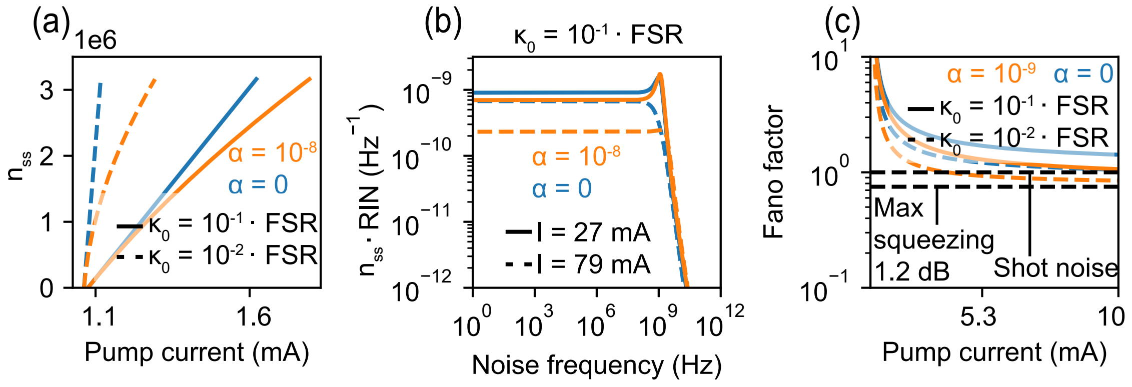

FIG. S2: **Steady state and noise plots for two photon absorption.** (a) Steady state intracavity photon number as a function of pump current (S-curve), demonstrating sub-linear dependence of photon number with pump current for two-photon absorption (TPA). (b) Photon number variance spectrum for two different pump strengths, with broadband squeezing for intensity-dependent TPA. (c) Fano factor plots for linear and TPA loss profiles. The intensity dependence of TPA $\kappa(n) \propto n$ creates small (< 2 dB) drops in Fano factor below the shot noise limit when pumped far above threshold. Here, $\alpha \equiv \alpha_{\text{TPA}}/\text{FSR}$.

D. Noise reduction using nonlinear distributed feedback-based loss

In this section, we consider distributed feedback semiconductor lasers where a distributed Bragg reflector (DBR) is fabricated on one (or both) ends/facets of the laser cavity, or a VCSEL-type structure is employed. In this case, we use the analytical form for DBR reflectivity given by coupled mode theory [S8, S9] to obtain

$$\kappa(\omega) = -\text{FSR} \cdot \log \left| \frac{g \sinh(\theta)}{\Gamma \cosh(\theta) + (\alpha_{\text{DBR}} + i\delta) \sinh(\theta)} \right|^2, \tag{S32}$$

where $\beta = \omega\tilde{n}/c$ is the propagation constant (wavevector), $g = \omega \Delta n_{\text{ref}}/(\pi c)$ is the approximate coupling coefficient, $\delta = \beta - \pi/d$, $\Gamma^2 = g^2 + (\alpha_{\text{DBR}} + i\delta)^2$, $\theta = N_{\text{DBR}} d\Gamma$, and α_{DBR} the radiative loss from the DBR. Here, N_{DBR} denotes the number of pairs of layers in the DBR, d the thickness of a pair of layers, Δn_{ref} the index contrast, $\tilde{n}$ the effective index, and $\omega \equiv \omega(n, N)$ the laser frequency. Note that δ has the interpretation of a detuning from the Bragg value π/d (the center of the Bragg stop-band of maximum reflectivity and thus lowest loss is at $\delta = 0$). We would like to operate in the "sharp loss" regime, which is where the stop-band switches over to a pass-band, first occurring when $\theta = \pi \implies \delta^2 - g^2 = \pi^2/L^2$. For a lossless DBR, choosing the frequency

ω_c at which this sharp transition occurs fixes δ and therefore Δn_{ref} from the above relations:

$$\Delta n_{\mathrm{ref}} = \frac{\pi c}{\omega_c}\sqrt{\left(\frac{\tilde{n}}{c}(\omega_c - \omega_t)\right)^2 - \left(\frac{\pi}{L}\right)^2}, \tag{S33}$$

where ω_t denotes the center of the stop band, so that $\omega_c - \omega_t$ is effectively the half-width of the stop band. The coupling coefficient g, index contrast Δn_{ref} and stop band width $2(\omega_t - \omega_c)$ are thus closely related.

To use Eq. S32, it is necessary to ensure the time response of the DBR is much faster than the free spectral range. We extract this time response by performing an FFT of $R(\omega)$. For lossy DBRs, $R(\omega)$ approaches a Lorentzian with width governed by α_{DBR}, and the maximum reflectivity may be far from unity. When the DBR is lossless, an analytical expression for the time response is in general difficult to obtain. We observe that the time response is faster for DBRs of larger bandgap (wider stop bands). Intuitively, outcoupling in a lossless DBR is through the coupling coefficient g which scales with the index contrast Δn_{ref} and thus correspondingly with the stop band width $2(\omega_c - \omega_t)$. This is distinct from the Fano resonances considered in the main text where the loss profile was derived from interference between a “direct channel” pathway bypassing the Fano resonance and an “indirect pathway” coupling to an intrinsic resonant mode of the photonic crystal. In such a case, the time response of the effective nonlinear dispersive loss is governed by the the complex resonance frequency of the Fano resonance (intuitively, how long light spends trapped in the photonic crystal). Here, however, sharply frequency-dependent loss arises from a different mechanism, namely the photonic bandgap of the DBR. A comparison of the two different types of temporal responses are provided in Fig. S3.

The sharpness of $\kappa(n)$ increases with the number of layers N_{DBR} and Kerr nonlinear strength (the former corresponds to sharper evanescent decay of modes in the photonic bandgap). For the strongest nonlinearity and sharpest $R(\omega)$, multiple stop bands may be accessible, corresponding to multiple regions of noise condensation and bistability for each transition from pass band to stop band. An important distinction from the Fano resonances considered earlier is that the sharp loss regions $\kappa_n > 0$ are now the lower bistability branches, accessible by simply pumping smoothly from threshold.

From an experimental standpoint, the sharpest loss (and strongest squeezing) can be obtained by maximizing the stop band width and number of layer pairs N_{DBR}. The former is limited by

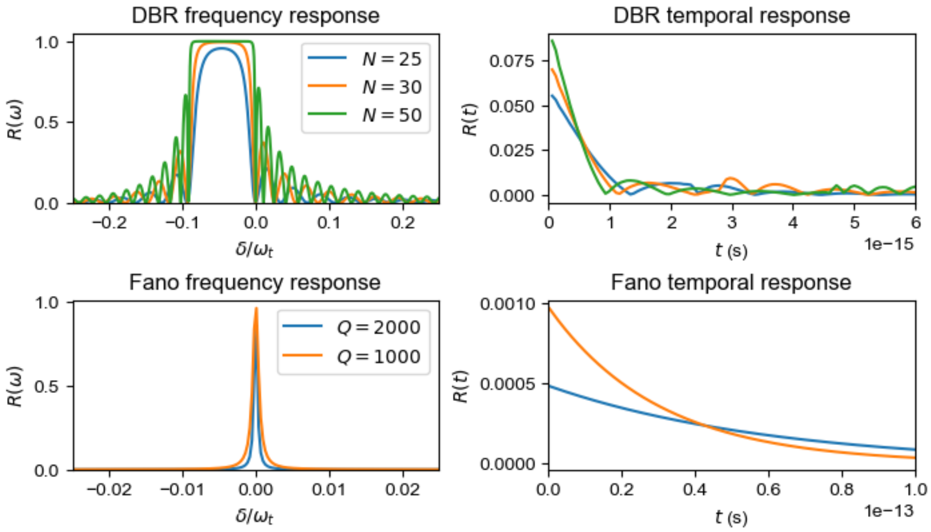

FIG. S3: **Comparison of frequency and temporal response for Fano mirror and DBR losses.** In the top row, the DBR stop band is made sharper and its width is increased by increasing the number of layers. This results in a shorter, ultrafast relaxation time. In contrast, in the bottom row, the Fano mirror frequency response is made sharper by increasing the quality factor (Q) of the resonance, which has the effect of decreasing the width of the resonance and increasing its lifetime.

the intracavity saturation intensity and required index contrast Δn_{ref}, while the latter is limited by fabrication methods. Nevertheless, carefully-engineered DBR-based losses when coupled to strong Kerr nonlinearity in semiconductor lasers may result in unprecedented broadband intensity noise squeezing, which we explore next.

1. Comparison of Fano mirror and DBR loss profiles

We now consider the noise properties of a Kerr nonlinear cavity with a distributed Bragg reflector (DBR) outcoupler. The loss profile (Fig. S4a) in this case marks a departure from the adiabaticity criterion that limits the sharpness of Fano-type losses because, as discussed in the previous section, its timescale is instead set by the width of the DBR pass/stop band, not the sharpness of its decay. In principle, this means that the DBR-type loss can be made quite large, enhancing intensity noise squeezing further. This is shown in Fig. S4b/c, where sharper loss profiles (obtained by increasing

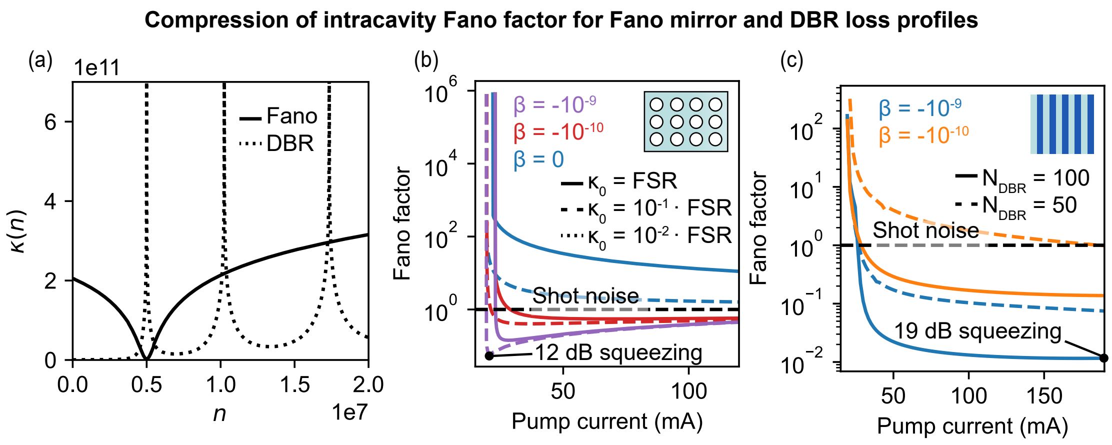

FIG. S4: **Comparison of loss profiles and integrated intracavity Fano factor as a function of pump current for a nonlinear laser with a Fano mirror or DBR.** (a) Loss profiles for a Kerr nonlinear cavity with outcoupling from a Fano mirror (solid) or DBR (dashed). (b) Intracavity Fano factor for the Fano loss profile as a function of pump current. (c) Intracavity Fano factor for the DBR loss profile as a function of pump current. In these simulations, $n_c = 5 \times 10^6$ marks the center of the Fano resonance, while for DBR loss profiles, the average index is $\tilde{n} = 3.0$, the index contrast is $\Delta n_{\text{ref}} \lesssim 1.0$, and the first transition from stop to pass band is tuned to occur around $n_c = 5 \times 10^6$. All other simulation parameters are the same for both architectures: cavity length $L = 1$ mm, cavity volume $V = 10^{-16}$ m^3, bare cavity resonance frequency $\omega_0 = 2.16 \times 10^{15}$ s^{-1}, transparency density $N_{\text{trans}} = 2 \times 10^{24}$ m^{-3}, nonradiative decay rate $\gamma_{||} = 3 \times 10^8$ s^{-1}, and linear gain coefficient $G_N = 1/V \cdot dG/dN = 3694$ s^{-1}.

the number of layers in the DBR) correspond to enhanced squeezing (over 5 dB lower than the Kerr-squeezed Fano laser example considered in the main text). Additionally, the sharp loss region ($\kappa_n > 0$) in the case of DBR loss profiles can be accessed by pumping directly from threshold, where a stop band transitions to a pass band. Multiple stop-pass band transitions are possible (three shown in Fig. S4a), corresponding to multiple bistable regions.

6. LINEWIDTH AND PHASE NOISE

The frequency noise spectrum and approximate linewidth are respectively given by [S1]

$$S_\phi(\Omega) = \langle \Omega^2 |\delta\hat{\phi}(\Omega)|^2 \rangle \tag{S34}$$

$$\Delta\omega \approx S_\phi(\Omega \to 0), \tag{S35}$$

where the linewidth can be approximated by evaluating the pole at $\Omega = 0$ in the intensity spectrum

$$I(\Omega) = \int_{-\infty}^{\infty} dt e^{-i\Omega t} \langle \hat{a}^\dagger(t)\hat{a}(0)\rangle, \tag{S36}$$

and assuming it is the dominant contribution to the residue (compared to the other poles near $\pm\Omega_R$, the relaxation oscillation frequency). For Fano resonance-based losses (as we consider in the main text), the adiabaticy criterion limits the sharpness of the loss κ_ω such that $\kappa_\omega \ll 1$ typically. For other types of dispersive losses, $\kappa_\omega \gg 1$ is possible, resulting in different frequency noise behaviors. We consider separately the cases of carrier-dependent and photon number-dependent shifts to the refractive index. In the former case, we find a linewidth

$$\left.\frac{\Delta\omega}{\Delta\omega_{\rm ST}}\right|_{\rm carrier} = \frac{1+\alpha_L^2}{(1-\alpha_L\kappa_\omega/2)^2} \tag{S37}$$

with $\Delta\omega_{\rm ST} = \kappa_{\rm ss}/(2n_{\rm ss})$ the Schawlow-Townes linewidth ($\kappa_{\rm ss} = G_{\rm ss}$ denotes the steady state loss (gain) and $n_{\rm ss}$ the steady state photon number). This result is consistent with that presented in earlier work using amplitude-phase coupling and dispersive loss to achieve linewidth reduction [S6, S10]. Notice that $\kappa_\omega \approx 2/\alpha_L$ results in linewidth broadening while $\kappa_\omega \gg 1$ can cause significant linewidth narrowing. In the case of dominant Kerr nonlinearity,

$$\left.\frac{\Delta\omega}{\Delta\omega_{\rm ST}}\right|_{\rm Kerr} = 1 + \frac{4}{\kappa_\omega^2}, \tag{S38}$$

thereby giving an effective linewidth enhancement factor $\alpha_{L,\rm eff} = 2/\kappa_\omega$. This shows linewidth broadening for $\kappa_\omega \ll 1$ and narrowing for $\kappa_\omega \gg 1$ (sharply dispersive losses).

For an estimate of the linewidth when it does not fall in these two limiting cases, we can use Sec. 5 B, finding

$$\begin{aligned}\langle \Omega^2|\delta\hat{\phi}(\Omega)|^2\rangle =& \langle 2\hat{D}_{\phi\phi}\rangle + \frac{\alpha^2 G_N^2}{4}\langle \delta\hat{N}^\dagger(\Omega)\delta\hat{N}(\Omega)\rangle + (\beta\omega_0)^2\langle \delta\hat{n}^\dagger(\Omega)\delta\hat{n}(\Omega)\rangle \\ &- \alpha G_N {\rm Re}\langle \hat{F}_\phi^\dagger \delta\hat{N}(\Omega)\rangle + 2\beta\omega_0 {\rm Re}\langle \hat{F}_\phi^\dagger \delta\hat{n}(\Omega)\rangle - \alpha G_N \beta\omega_0 {\rm Re}\langle \delta\hat{N}^\dagger(\Omega)\delta\hat{n}(\Omega)\rangle.\end{aligned} \tag{S39}$$

In Fig. S5, we plot the phase noise spectrum and calculate the Fano factor and effective linewidth enhancement factor $\alpha_{L,\rm eff}$ for two lasers, one without and the other with nonlinear dissipation. The latter features 14 dB squeezing in intensity noise, with a 6X increase in the effective linewidth enhancement factor. The approximate linewidth is calculated as

$$\Delta\nu = \frac{1}{2\pi}\frac{R_{sp}}{2n_{\rm ss}}(1+\alpha_{L,\rm eff}^2). \tag{S40}$$

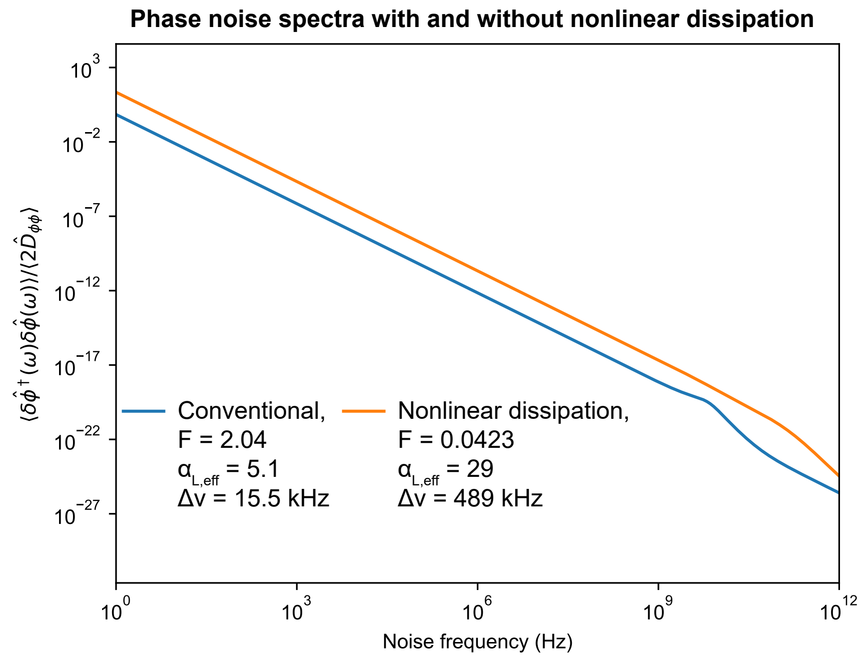

FIG. S5: **Phase noise spectra for laser systems with and without nonlinear dissipation.** The laser with nonlinear dissipation features 14 dB intensity noise squeezing in the Fano factor F and an approximately 6X increase in the effective linewidth enhancement factor $\alpha_{L,\text{eff}}$. The approximate linewidth calculated by Eq. S35 is denoted by $\Delta\nu = 2\pi\Delta\omega$. Simulation parameters: $G_N = 10^4$ s^{-1}, $\alpha_L = 5.0$, $\gamma = 2 \times 10^{12}$ s^{-1}, $\beta = -10^{-9}$, $n_c = 5 \times 10^6$, FSR $= 3.0 \times 10^{11}$ Hz. Both curves are evaluated at the same photon number $n = 5.5 \times 10^6$ and the steady state loss is equal in both cases.

7. OUTPUT PHOTON NOISE IN LASERS WITH NONLINEAR DISPERSIVE LOSS

Here, we develop formalism to compute output photon noise in the presence of nonlinearity and dispersive dissipation. Let a, d respectively denote the nonlinear cavity mode and the Fano mirror mode, both of which couple to a continuum of far-field modes s_k (here k labels momentum). From the full Hamiltonian of the system [S11], the Heisenberg equations of motion can be derived as

$$\begin{aligned}
\dot{\hat{a}} &= -i\omega_a(1 + \beta\hat{a}^\dagger\hat{a} + \sigma\Delta\hat{N})\hat{a} + G(\hat{N})(1 - i\alpha_L)\hat{a} - i\sum_k g_k^*\hat{s}_k + \hat{F}_G \\
\dot{\hat{d}} &= -i\omega_d\hat{d} - i\sum_k v_k^*\hat{s}_k \\
\dot{\hat{s}}_k &= -i\omega_k\hat{s}_k - i(g_k\hat{a} + v_k\hat{d}).
\end{aligned} \tag{S41}$$

where $\Delta\hat{N} \equiv \hat{N} - N_{\text{trans}}$ indicates the free carrier population and $\omega_{a,d}$ denote the resonance frequencies of the cavity and Fano mirror, β is the per-photon Kerr nonlinearity, $G(\hat{N})$ is the carrier-dependent gain, α_L is the linewidth enhancement factor, and g_k, v_k are the couplings of $\hat{a}, \hat{d}$ to the far-field mode $\hat{s}_k$. $\hat{F}_G(t)$ is a Langevin force term for the gain. We neglect direct coupling between $\hat{a}$ and $\hat{d}$, though this can be readily incorporated into the Heisenberg equations. We can solve for $\hat{s}_k$ as

$$\hat{s}_k(t) = \hat{s}_k(0)e^{-i\omega_k t} - i\int^t dt' e^{-i\omega_k(t-t')}[g_k\hat{a}(t') + v_k\hat{d}(t')]. \tag{S42}$$

We assume momentum independent coupling $g = g_k, v = v_k$ and introduce $\kappa_{\text{FSR}} = \pi\rho|g|^2, \gamma = \pi\rho|v|^2$. The input-output relation can then be found by taking $t \to \infty$, performing an integral over k, and Fourier transforming:

$$\hat{s}_{\text{out}}(\omega) = -\hat{s}_{\text{in}}(\omega) + \sqrt{2\kappa_{\text{FSR}}}\hat{a}(\omega) + \sqrt{2\gamma}\hat{d}(\omega). \tag{S43}$$

We can write the Fourier transformed Heisenberg equations for a, d as

$$\begin{aligned} -i\omega\hat{a} &= -i\omega_a(1 + \beta\hat{a}^\dagger\hat{a} + \sigma\Delta\hat{N})\hat{a} + [G(\hat{N})(1 - i\alpha_L) - \kappa_{\text{FSR}}]\hat{a} - \sqrt{\kappa_{\text{FSR}}\gamma}\hat{d} + \sqrt{2\kappa_{\text{FSR}}}\hat{s}_{\text{in}} + \hat{F}_G \\ -i\omega\hat{d} &= -i\omega_d\hat{d} - \gamma\hat{d} - \sqrt{\kappa_{\text{FSR}}\gamma}\hat{a} + \sqrt{2\gamma}\hat{s}_{\text{in}}. \end{aligned} \tag{S44}$$

Eliminating $\hat{d}(\omega)$ as

$$d(\omega) = \frac{\sqrt{2\gamma}\hat{s}_{\text{in}} - \sqrt{\kappa_{\text{FSR}}\gamma}\hat{a}}{i\delta_d + \gamma}, \tag{S45}$$

with $\delta_d \equiv \omega_d - \omega$, we can write the Fourier transformed equation of motion for a and the input-output relation as

$$-i\omega\hat{a} = -i\omega_a(1 + \beta\hat{a}^\dagger\hat{a} + \sigma\Delta\hat{N})\hat{a} + [G(\hat{N})(1 - i\alpha_L) - K_l(\omega)]\hat{a} + \overbrace{K_c(\omega)\hat{s}_{\text{in}} + \hat{F}_G}^{\hat{F}_a} \tag{S46}$$

$$\hat{s}_{\text{out}}(\omega) = K_a(\omega)\hat{a}(\omega) - K_s(\omega)\hat{s}_{\text{in}}(\omega), \tag{S47}$$

where

$$\begin{aligned} K_a(\omega) &= \sqrt{2\kappa_{\text{FSR}}}\left(1 - \frac{\gamma}{i\delta_d + \gamma}\right) \\ K_s(\omega) &= 1 - \frac{2\gamma}{i\delta_d + \gamma} \\ K_l(\omega) &= \kappa_{\text{FSR}}\left(1 - \frac{\gamma}{i\delta_d + \gamma}\right) \\ K_c(\omega) &= K_a(\omega). \end{aligned} \tag{S48}$$

To compute noise, we begin with the intracavity fluctuations, which are governed by the linearized system

$$M(\Omega)\begin{bmatrix}\delta\hat{a}(\Omega)\\ \delta\hat{a}^\dagger(\Omega)\\ \delta\hat{N}(\Omega)\end{bmatrix}=\begin{bmatrix}\hat{F}_a(\Omega)\\ \hat{F}_{a^\dagger}(\Omega)\\ \hat{F}_N(\Omega)\end{bmatrix}=\begin{bmatrix}K_c(\omega_+)\delta\hat{s}_{\text{in}}(\Omega)+\hat{F}_G(\Omega)\\ K_c^*(\omega_-)\delta\hat{s}_{\text{in}}^\dagger(\Omega)+\hat{F}_G^\dagger(\Omega)\\ \hat{F}_N(\Omega)\end{bmatrix}, \tag{S49}$$

where for a generic operator $\hat{X}^\dagger(\Omega)=[\hat{X}(-\Omega)]^\dagger$ follows from the definition $\hat{X}^\dagger(t)=[\hat{X}(t)]^\dagger$. The fluctuation matrix has columns

$$\begin{aligned}
M_{x1}(\Omega)&=\begin{bmatrix}-i\omega_+ + i\omega_a(1+2\beta|\alpha|^2_{\text{ss}}+\sigma\Delta N_{\text{ss}})+K_l(\omega_+)-G(N_{\text{ss}})(1-i\alpha_L)\\ -i\omega_a\beta\alpha^{*2}_{\text{ss}}\\ 2G(N_{\text{ss}})\alpha^*_{\text{ss}}\end{bmatrix}\\
M_{x2}(\Omega)&=\begin{bmatrix}i\omega_a\beta\alpha^2_{\text{ss}}\\ i\omega_- - i\omega_a(1+2\beta|\alpha|^2_{\text{ss}}+\sigma\Delta N_{\text{ss}})+K_l^*(\omega_-)-G(N_{\text{ss}})(1+i\alpha_L)\\ 2G(N_{\text{ss}})\alpha_{\text{ss}}\end{bmatrix}\\
M_{x3}(\Omega)&=\begin{bmatrix}-G_N\alpha_{\text{ss}}(1-i\alpha_L)+i\omega_a\sigma\alpha_{\text{ss}}\\ -G_N\alpha_{\text{ss}}(1+i\alpha_L)-i\omega_a\sigma\alpha^*_{\text{ss}}\\ -i\omega_{\text{ss}}+\gamma_\parallel+2G_N|\alpha|^2_{\text{ss}}\end{bmatrix}.
\end{aligned} \tag{S50}$$

Here, $x\in[1,2,3]$ to denote the row of M, $\omega_\pm=\omega_{\text{ss}}\pm\Omega$, and the steady state cavity amplitude α_{ss} is determined through

$$[i(-\omega_a(1+\beta|\alpha|^2_{\text{ss}}+\sigma\Delta N_{\text{ss}})+\omega)+G(N_{\text{ss}})(1-i\alpha_L)-K_l(\omega_{\text{ss}})]\alpha_{\text{ss}}=0. \tag{S51}$$

Noise emerges from the nonzero correlators [S1, S12]

$$\begin{aligned}
\langle\hat{F}_G^\dagger(\omega)\hat{F}_G(\omega')\rangle&=G(N)\delta(\omega-\omega')\\
\langle\hat{F}_N^\dagger(\omega)\hat{F}_G(\omega')\rangle&=-\alpha G(N)\delta(\omega-\omega')\\
\langle\hat{F}_N^\dagger(\omega)\hat{F}_N(\omega')\rangle&=[nG(N)+\gamma_\parallel N+\epsilon I]\delta(\omega-\omega')\\
\langle\hat{s}_{\text{in}}(\omega)\hat{s}_{\text{in}}^\dagger(\omega')\rangle&=\delta(\omega-\omega'),
\end{aligned} \tag{S52}$$

with $\epsilon=0$ for quiet pumping and $\epsilon=1$ for shot noise limited pumping. We can now compute the

output photon noise by noting

$$\begin{aligned}\delta\hat{n}_{\text{out}}(t) &= s_0^*\delta\hat{s}_{\text{out}}(t) + s_0\delta\hat{s}_{\text{out}}^\dagger(t) \\ \delta\hat{n}_{\text{out}}(\Omega) &= s_0^*\delta\hat{s}_{\text{out}}(\Omega) + s_0[\delta\hat{s}_{\text{out}}(-\Omega)]^\dagger \\ &= s_0^*K_a(\omega_+)\delta\hat{a}(\Omega) + s_0K_a^*(\omega_-)\delta\hat{a}^\dagger(\Omega) - [s_0^*K_s(\omega_+)\delta\hat{s}_{\text{in}}(\Omega) + s_0K_s^*(\omega_-)\delta\hat{s}_{\text{in}}^\dagger(\Omega)]\end{aligned} \tag{S53}$$

where here $s_0(\omega) = K_a(\omega)\alpha_{\text{ss}}(\omega)$ is the steady state output (propagating) amplitude and the intensity noise spectrum is given by $\langle\delta\hat{n}_{\text{out}}^\dagger(\Omega)\delta\hat{n}_{\text{out}}(\Omega)\rangle$. Spectra in the limit of nondispersive loss closely match those found by Yamamoto et al. [S12].

8. INTENSITY NOISE IN QCLS WITH NONLINEAR DISPERSIVE LOSS

The photon and carrier dynamics for QCLs are conventionally described using a three-level model for the carrier populations [S13]

$$\begin{aligned}\dot{N}_3^j &= I^j - N_3^j\left(\frac{1}{\tau_{32}} + \frac{1}{\tau_{31}}\right) - nG\left(N_3^j, N_2^j\right) \\ \dot{N}_2^j &= \frac{N_3^j}{\tau_{32}} - \frac{N_2^j}{\tau_{21}} + nG\left(N_3^j, N_2^j\right) \\ \dot{n} &= n\left(-\kappa(n) + \sum_{j=1}^m G\left(N_3^j, N_2^j\right)\right),\end{aligned} \tag{S54}$$

where N_3^j, N_2^j respectively denote the carrier populations in levels 3 and 2 in each gain stage j, I^j denotes the injected current to gain stage j, and $\tau_{31}, \tau_{32}, \tau_{21}$ are the nonradiative decay time constants indicated in Fig. 4c. A linear model for the gain $G\left(N_3^j, N_2^j\right) = G_N(N_3^j - N_2^j)$ is employed. Langevin forces $\hat{F}_n^j, \hat{F}_3^j, \hat{F}_2^j$ are added to the respective mean field equations of motion for the following noise analysis.

We can simplify the analysis by introducing $N_{2,3} = \sum_j N_{3,2}^j$ and assuming all of the gain stages are identical. Then, the dynamics for n, N_2, N_3 are described by a set of three coupled nonlinear equations. Note that we neglect the dynamics of N_1 (the carrier population in level 1) since the populations of interest n, N_2, N_3 form a closed system of equations. Linearizing and Fourier

transforming the QCL rate equations, we find

$$M \begin{bmatrix} \delta\hat{N}_3(\Omega) \\ \delta\hat{N}_2(\Omega) \\ \delta\hat{n}(\Omega) \end{bmatrix} = \begin{bmatrix} \hat{F}_3 \\ \hat{F}_2 \\ \hat{F}_n - n_{\mathrm{ss}}\kappa_\omega \hat{F}_\phi \end{bmatrix}$$

with the fluctuation matrix

$$M = \begin{bmatrix} -i\Omega + \gamma_{11} & -\gamma_{12} & \gamma_{13} \\ -\gamma_{21} & -i\Omega + \gamma_{22} & -\gamma_{23} \\ -\gamma_{31} & \gamma_{32} & -i\Omega - \gamma_{33} \end{bmatrix},$$

where $\gamma_{11} = n_{\mathrm{ss}}G_N + 1/\tau_{32} + 1/\tau_{31}, \gamma_{12} = n_{\mathrm{ss}}G_N, \gamma_{13} = \gamma_{23} = G_N\Delta N_{\mathrm{ss}}, \gamma_{21} = n_{\mathrm{ss}}G_N + 1/\tau_{32}, \gamma_{22} = n_{\mathrm{ss}}G_N + 1/\tau_{21}, \gamma_{31} = \gamma_{32} = n_{\mathrm{ss}}G_N, \gamma_{33} = -n_{\mathrm{ss}}\kappa_n$ and $\Delta N = N_3 - N_2$. The correlators between the Langevin forces are given by $\langle 2\hat{D}_{nn}\rangle = 2G_N N_3 n$, $\langle 2\hat{D}_{\phi\phi}\rangle = G_N N_3/(2n)$, $\langle 2\hat{D}_{22}\rangle = 2G_N N_3 n + N_3/\tau_{32}$, $\langle 2\hat{D}_{33}\rangle = 2G_N N_3 n + N_3/\tau_{32} + N_3/\tau_{31}$, $\langle 2\hat{D}_{3n}\rangle = -G_N\left(N_2 + N_3\right)n$, $\langle 2\hat{D}_{2n}\rangle = G_N\left(N_2 + N_3\right)n$, $\langle 2\hat{D}_{32}\rangle = -\left(G_N(N_2 + N_3)n + N_3/\tau_{32}\right)$.

In QCLs, the intensity noise is dominated by both spontaneous emission and nonradiative decay of excited carriers, whereas in typical semiconductor lasers, it is only the former that matters [S14]. Thus, starting from the linearized matrix equations, we can approximate the DC intensity noise as

$$\langle \delta\hat{n}^\dagger(\Omega = 0)\delta\hat{n}(\Omega = 0)\rangle \approx \frac{\gamma_s^2(\gamma_{21} - \gamma_{22})^2\langle 2\hat{D}_{33}\rangle + (\gamma_s\gamma_{21} - \gamma_{11}\gamma_{22})^2\langle 2\hat{D}_{nn}\rangle}{\left(\gamma_s^2\gamma_{23} + \gamma_{11}\gamma_{22}\gamma_{33} + \gamma_s(\gamma_{13}(\gamma_{21} - \gamma_{22}) - \gamma_{11}\gamma_{23} = \gamma_{21}\gamma_{33})\right)^2}, \tag{S55}$$

where $\gamma_s = \gamma_{12} = \gamma_{31} = \gamma_{32} = n_{\mathrm{ss}}G_N$. In the absence of nonlinear dispersive loss, $\gamma_{33} = 0$ and the DC intensity noise goes as $(\tau_s/\tau_{\mathrm{nr}})^2$ where $1/\tau_s \equiv n_{\mathrm{ss}}G_N$ is the rate of stimulated emission (per carrier) and $1/\tau_{\mathrm{nr}}$ is an effective nonradiative decay rate of the carriers. The scaling with the stimulated emission lifetime is expected given that the light approaches a coherent state as the power increases. The inverse scaling with τ_{nr} reflects the fact that in QCLs, in contrast to conventional semiconductor lasers, the carrier density is not clamped above threshold. Instead, N_2, N_3 are dynamic and their fluctuations have fast response times, significantly affecting the intensity noise even above threshold. We also note that the fast nonradiative decay of the carriers also leads to the relaxation oscillations in QCLs being overdamped, despite increasing intensity noise. In this case, the increased intensity noise of QCLs compared to conventional lasers stems from stronger low-frequency noise arising from the unclamped carrier populations above threshold

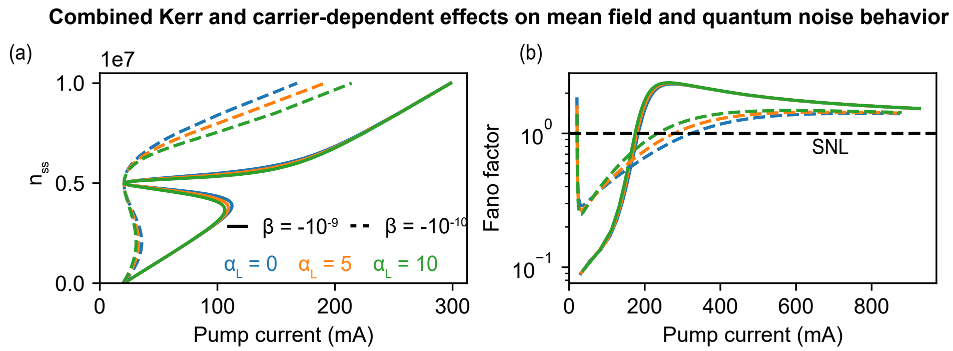

FIG. S6: **Effects of carrier and Kerr nonlinearities composed with dispersive loss**. (a) Bistable input-output curves for two different Kerr nonlinearity strengths β and three different carrier nonlinearity strengths (controlled by the linewidth enhancement factor α_L). (b) Integrated intracavity Fano factor corresponding to the different Kerr and carrier nonlinearity strengths. System parameters are the same as those considered in Figs. 2/3 of the main text.

(which increase proportionately with pump current, together with the photon number). The effect of nonlinear dispersive loss is to outcompete the nonradiative decay rates to dominate the intensity noise. Thus, $|\gamma_{33}| \gg 1/\tau_{\rm nr}$ is a necessary condition for this mechanism for squeezing to be effective.

To provide analytical checks against previous theory on QCL intensity noise [S13], we compute output photon noise as described in the Methods section of the main manuscript, agreeing qualitatively with Eq. 95 of [S13].

9. NONLINEAR DISPERSIVE LOSS WITH CARRIER AND KERR NONLINEARITIES

In the main text, it was noted that carrier nonlinearity could be reasonably neglected for the example systems considered. In this section, we show that this is indeed the case by studying the effect of nonzero linewidth enhancement α_L (which controls the carrier nonlinearity σ). We simulate the same semiconductor laser systems considered in Figs. 2/3 of the main text, now with varying α_L. In Fig. 8, we show the effects of carrier nonlinearity on the steady state input-output curve (photon number as a function of pump current) and quantum noise (intracavity integrated

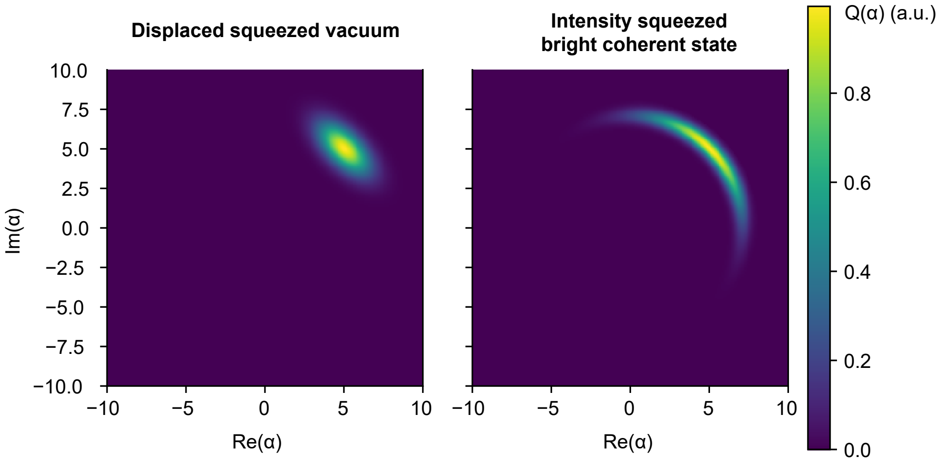

FIG. S7: **Schematic illustration of Husimi Q function of few photon intracavity quantum state under nonlinear dissipation.** Left: displaced quadrature squeezed vacuum, right: direct intensity (photon number) squeezing of a bright coherent state. $Q(\alpha)$ denotes the Husimi Q function.

Fano factor). As shown, carrier nonlinearity only becomes important when Kerr nonlinearity is weak or absent. In particular, notice that maximum squeezing is achieved near the leftmost tip of the upper bistable branch, which lies close to threshold (for the low loss architectures necessary for our scheme). Around this point, the free carrier population is relatively small (low gain) and has little impact on the intensity noise through carrier-dependent shifts to the refractive index.

10. QUANTUM PHASE SPACE DISTRIBUTION IN SYSTEMS WITH NONLINEAR DISSIPATION

The phase space distribution of the intracavity quantum state produced under nonlinear dissipation can be rigorously calculated using density matrix formalism. At macroscopic intensities (which is the case in our work, since we consider intracavity photon numbers in excess of 10^6), the quantum states in the systems we consider can be well approximated as Gaussian. This is the basis for using the linearized Heisenberg-Langevin equation formalism, where the Langevin noise sources are Gaussian and only their second-order moments are necessary for characterizing the Gaussian noise distribution. In Fig. S7, we show a schematic illustration for an example of what the intracavity quantum state would look like in the few photon/mesoscopic regime, depicted through its Husimi Q function, $Q(\alpha) = \frac{1}{\pi} \langle\alpha| \hat{\rho} |\alpha\rangle$ (with $|\alpha\rangle$ a coherent state and $\hat{\rho}$ the system's density matrix restricted to its photonic degrees of freedom, with all other degrees of freedom

traced out). Importantly, because nonlinear dissipation squeezes the state directly in photon number rather than displacing squeezed vacuum, the Husimi Q function would feature squeezed radial width and extend circumferentially. In contrast, displaced squeezed vacuum could have the same center point, but the distribution would look like an elongated ellipse with major axis along the antisqueezed quadrature. This difference becomes significant in the few photon and mesoscopic regimes, where nonlinear dissipation offers a route to create highly intensity squeezed states approaching multi-photon Fock states, which is inaccessible with other forms of squeezing.

[S1] Weng W Chow, Stephan W Koch, and Murray III Sargent. *Semiconductor-laser physics*. Springer Science & Business Media, 2012.

[S2] Y Yamamoto, N Imoto, and S Machida. Amplitude squeezing in a semiconductor laser using quantum nondemolition measurement and negative feedback. *Physical Review A*, 33(5):3243, 1986.

[S3] Yi Yu, Weiqi Xue, Elizaveta Semenova, Kresten Yvind, and Jesper Mork. Demonstration of a self-pulsing photonic crystal fano laser. *Nature Photonics*, 11(2):81–84, 2017.

[S4] PD Drummond and DF Walls. Quantum theory of optical bistability. i. nonlinear polarisability model. *Journal of Physics A: Mathematical and General*, 13(2):725, 1980.

[S5] Robert W Boyd. *Nonlinear optics*. Academic press, 2020.

[S6] Rashit Nabiev, Yuri Popov, and Amnon Yariv. Semiconductor laser with dispersive loss: Quantum noises and amplitude squeezing. *Journal de Physique III*, 2(9):1605–1614, 1992.

[S7] Michael A Newkirk and Kerry J Vahala. Amplitude-phase decorrelation: a method for reducing intensity noise in semiconductor lasers. *IEEE journal of quantum electronics*, 27(1):13–22, 1991.

[S8] Luigi R Brovelli and Ursula Keller. Simple analytical expressions for the reflectivity and the penetration depth of a bragg mirror between arbitrary media. *Optics communications*, 116(4-6):343–350, 1995.

[S9] HW Yen, W Ng, I Samid, and A Yariv. Gaas distributed bragg reflector lasers. *Optics Communications*, 17(3):213–218, 1976.

[S10] Amnon Yariv, Rashit Nabiev, and Kerry Vahala. Self-quenching of fundamental phase and amplitude noise in semiconductor lasers with dispersive loss. *Optics letters*, 15(23):1359–1361, 1990.

[S11] Nicholas Rivera, Jamison Sloan, Yannick Salamin, John D Joannopoulos, and Marin Soljačić. Creating large fock states and massively squeezed states in optics using systems with nonlinear bound

states in the continuum. *Proceedings of the National Academy of Sciences*, 120(9):e2219208120, 2023.

[S12] YOSHIHISA Yamamoto and NOBUYUKI Imoto. Internal and external field fluctuations of a laser oscillator: Part i–quantum mechanical langevin treatment. *IEEE journal of quantum electronics*, 22(10):2032–2042, 1986.

[S13] Farhan Rana and Rajeev J Ram. Current noise and photon noise in quantum cascade lasers. *Physical Review B*, 65(12):125313, 2002.

[S14] Tobias Gensty, Wolfgang Elsäßer, and Christian Mann. Intensity noise properties of quantum cascade lasers. *Optics Express*, 13(6):2032–2039, 2005.
